# Omega 3 fatty acids chemosensitize multidrug resistant colon cancer cells by down-regulating cholesterol synthesis and altering detergent resistant membranes composition

**DOI:** 10.1186/1476-4598-12-137

**Published:** 2013-11-13

**Authors:** Giada Gelsomino, Paola A Corsetto, Ivana Campia, Gigliola Montorfano, Joanna Kopecka, Barbara Castella, Elena Gazzano, Dario Ghigo, Angela M Rizzo, Chiara Riganti

**Affiliations:** 1 Department of Oncology, University of Torino, via Santena 5/bis, 10126 Torino, Italy; 2 Department of Pharmacological and Biomolecular Sciences, University of Milano, via Trentacoste 2, 20134 Milan, Italy; 3 Center for Experimental Research and Medical Studies, University of Torino, via Santena 5/bis, 10126 Torino, Italy

**Keywords:** Omega 3 polyunsaturated fatty acids, Cholesterol, Detergent resistant membranes, Multidrug resistance, ATP binding cassette transporters

## Abstract

**Background:**

The activity of P-glycoprotein (Pgp) and multidrug resistance related protein 1 (MRP1), two membrane transporters involved in multidrug resistance of colon cancer, is increased by high amounts of cholesterol in plasma membrane and detergent resistant membranes (DRMs). It has never been investigated whether omega 3 polyunsatured fatty acids (PUFAs), which modulate cholesterol homeostasis in dyslipidemic syndromes and have chemopreventive effects in colon cancer, may affect the response to chemotherapy in multidrug resistant (MDR) tumors.

**Methods:**

We studied the effect of omega 3 PUFAs docosahexaenoic acid (DHA) and eicosapentaenoic acid (EPA) in human chemosensitive colon cancer HT29 cells and in their MDR counterpart, HT29-dx cells.

**Results:**

MDR cells, which overexpressed Pgp and MRP1, had a dysregulated cholesterol metabolism, due to the lower expression of ubiquitin E3 ligase Trc8: this produced lower ubiquitination rate of 3-hydroxy-3-methylglutaryl-coenzyme A reductase (HMGCoAR), higher cholesterol synthesis, higher cholesterol content in MDR cells. We found that DHA and EPA re-activated Trc8 E3 ligase in MDR cells, restored the ubiquitination rate of HMGCoAR to levels comparable with chemosensitive cells, reduced the cholesterol synthesis and incorporation in DRMs. Omega 3 PUFAs were incorporated in whole lipids as well as in DRMs of MDR cells, and altered the lipid composition of these compartments. They reduced the amount of Pgp and MRP1 contained in DRMs, decreased the transporters activity, restored the antitumor effects of different chemotherapeutic drugs, restored a proper tumor-immune system recognition in response to chemotherapy in MDR cells.

**Conclusions:**

Our work describes a new biochemical effect of omega 3 PUFAs, which can be useful to overcome chemoresistance in MDR colon cancer cells.

## Background

Omega 3 polyunsaturated fatty acids (ω3PUFAs), such as docosahexaenoic acid (DHA) and eicosapentaenoic acid (EPA), have been reported in the last years to exert significant benefits in cardiovascular diseases (where they limit the formation of atherosclerotic plaque, decrease the production of pro-inflammatory cytokines, reduce the recruitment of leukocytes to the arterial wall [1]), inflammatory diseases and cancer [2]. For instance ω3PUFAs have been proposed as chemopreventive agents or adjuvant agents in association with radiotherapy and chemotherapy in specific tumors [3]. Despite the high number of studies reporting a significant reduction in the cardiovascular risk for patients treated with ω3PUFA supplements, some contrasting data about the benefits of ω3PUFAs in cardiovascular diseases have emerged recently [4-6]. Also the chemopreventive effects of ω3PUFAs have been re-discussed in the light of recent experimental works, showing a potential positive correlation between serum levels of DHA and/or EPA and increased incidence of prostate cancer [7-10].

Worth of note colon cancer is a diet-related cancer: a diet rich of saturated fatty acids is an established risk factor for the onset of colon tumors, whereas ω3PUFA supplementation lowers the incidence of this malignancy [11]. The chemopreventive role of ω3PUFAs in colon cancer is supported by experimental observations showing that EPA supplementation reduces the size and number of polyps, lowers colon cell proliferation, increases apoptosis in mice harboring APC mutations [12] and in patients with a previous history of colon adenomas [13]. *In vitro* studies suggest that the anti-proliferative effect of ω3PUFAs can be due to increased production of reactive oxygen species [14], increased DNA strand breaks and cell cycle arrest [15], and changes in proteins involved in apoptosis, detoxification and cell cycle control [16].

One of the most interesting metabolic effects of EPA and DHA is their positive impact on cholesterol homeostasis: in dyslipidemic rats a diet enriched with PUFAs, including ω3PUFAs, favors the reverse cholesterol transport and increases high density lipoprotein (HDL) cholesterol [17]; in humans, ω3FA supplementation decreases triglycerides but also increases both HDL and low density lipoprotein (LDL) cholesterol [18,19]. Only few works highlighted a direct effect of ω3PUFAs on 3-hydroxy-3-methylglutaryl-coenzyme A reductase (HMGCoAR), the enzyme which catalyzes the limiting step of the cholesterol biosynthesis. In rats, ω3PUFA supplementation prevents the age-related activation of HMGCoAR in liver, by maintaining the enzyme constantly phosphorylated on serine and inhibited [20]. EPA and DHA reduce HMGCoAR activity in MCF-7 human breast cancer cells [21]. In colon cancer cells, the effects of ω3PUFAs are more controversial: for instance, EPA inhibits HMGCoAR activity in CaCo-2 colon cancer cells [22]; DHA has little effect on the *de novo* synthesis of cholesterol in SW620 cells [23], although it increases the sterol regulatory element binding protein-2 (SREBP-2), which induces the transcription of several genes involved in the cholesterol synthesis - e.g. HMGCoAR and 3-hydroxy-3-methylglutaryl-coenzyme A synthase (HMGCoAS) [23,24].

We recently showed that a high rate of cholesterol synthesis in colon cancer is critical to produce the phenotype known as multidrug resistance (MDR), a condition that makes cells simultaneously unresponsive to different drugs, unrelated for chemical structure and mechanism of action [25,26]. One of the main mechanisms of MDR is the overexpression of membrane ATP binding cassette (ABC) transporters, such as P-glycoprotein (Pgp/ABCB1), multidrug resistance related proteins (MRPs/ABCCs), breast cancer resistance protein (BCRP/ABCG2). By inducing the efflux of chemotherapeutic drugs, ABC transporters limit the intracellular accumulation and toxicity of several anticancer agents [27]. The activity of Pgp [28] and BCRP [29,30] is directly related to the amount of cholesterol in the plasma membrane. Chemoresistant HT29-dx colon cancer cells have higher levels of HMGCoAR [25] and greater amounts of membrane cholesterol [25,26] than chemosensitive HT29 cells. Drugs lowering the endogenous synthesis of cholesterol – e.g. statins and aminobisphosphonates – decrease Pgp activity [25] and expression [26] in HT29-dx colon cancer cells, increasing their chemosensitivity to Pgp substrates. A significant fraction of Pgp, MRP1 and BCRP is embedded in cholesterol-rich domains of the plasma membrane, such as detergent resistant membranes (DRMs) [29-31]. Indeed, the onset of MDR in cancer is paralleled by a progressive enrichment of cholesterol in DRMs [32].

Of note, ω3PUFAs can be incorporated in plasma membrane [33] and DRMs [34-36], where they increase the degree of lipid unsaturation, alter the physicochemical properties of these compartments (e.g. by displacing cholesterol) and impair the functional activity of several DRM-associated proteins [34,36,37]. Due to its high poly-unsaturation and to its longer carbon chain, DHA is poorly compatible with the ordered cholesterol/glycosphingolipids disposition in DRMs, and it is a stronger DRM-disrupting agent than EPA [34].

To our knowledge, there is no information on the effects exerted by ω3PUFAs on cholesterol metabolism, DRMs composition, activity of ABC transporters and MDR phenotype in colon cancer.

In this work we investigated: 1) whether and how ω3PUFAs modulate the synthesis of cholesterol and the composition of DRMs in human chemosensitive and chemoresistant colon cancer cells; 2) whether they impair the activity of ABC transporters, inducing chemosensitization. To this aim we treated human chemosensitive colon cancer HT29 cells and their resistant counterpart (HT29-dx cells) with EPA and DHA; to verify the specificity of ω3PUFA effects, we compared the effects of EPA and DHA with those of the omega 6 polyunsaturated fatty acid (ω6PUFA) arachidonic acid (AA).

## Results

### ω3PUFAs reduce the de novo cholesterol synthesis in colon cancer cells

**Figure 1:**
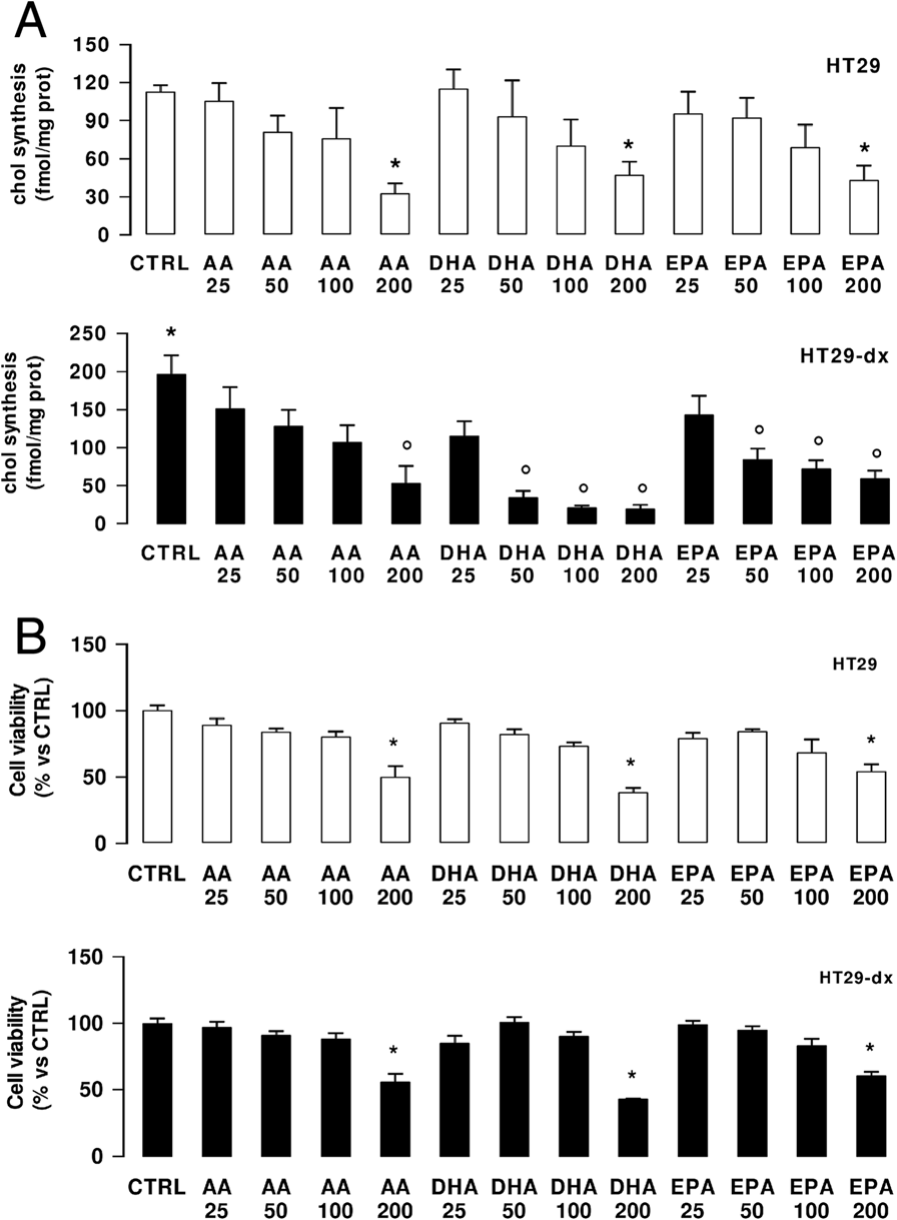
**Effects of ω6PUFAs and ω3PUFAs on cholesterol synthesis and cells viability in colon cancer cells.** HT29 and HT29-dx cells were incubated for 24 h in the absence (CTRL) or in the presence of various concentrations (25, 50, 100, 200 μM) of arachidonic acid (AA), docosahexaenoic acid (DHA), eicosapentaenoic acid (EPA) and subjected to the following investigations. **(A)** Cells were grown in a medium containing [^3^H]acetate, then the *de novo* synthesis of cholesterol was measured in duplicate as described in Methods. Data are presented as means ± SD (n = 4). Versus CTRL HT29 cells: * p < 0.05; versus CTRL HT29-dx cells: ° p < 0.05. **(B)** Cells were stained with Neutral Red solution and the absorbance of viable cells was measured in triplicate spectrophotometrically. Data are presented as means ± SD (n = 4). Versus respective CTRL: * p < 0.05.

Chemoresistant HT29-dx colon cancer cells exhibited a significantly higher synthesis of cholesterol when compared to chemosensitive HT29 cells (Figure 1A). The ω6PUFA AA did not affect the cholesterol synthesis in both cell lines, except at 200 μM AA; ω3PUFAs DHA and EPA decreased the cholesterol synthesis starting from 50 μM. Such decrease was more pronounced and statistically significant in HT29-dx cells (Figure 1A).

All PUFAs reduced the viability of both cell lines at 200 μM (Figure 1B); at 50 μM they did not impair cell viability (Figure 1B), did not induce apoptosis (Additional file 1A) and necrotic/immunogenic death (Additional file 1B).

Since 50 μM PUFA was the lowest concentration able to decrease the endogenous synthesis of cholesterol in chemoresistant cells without exerting toxicity *per se*, it was chosen for all the subsequent experiments.

### ω3PUFAs reduce HMGCoAR expression in colon chemoresistant cancer cells by increasing its ubiquitination

**Figure 2:**
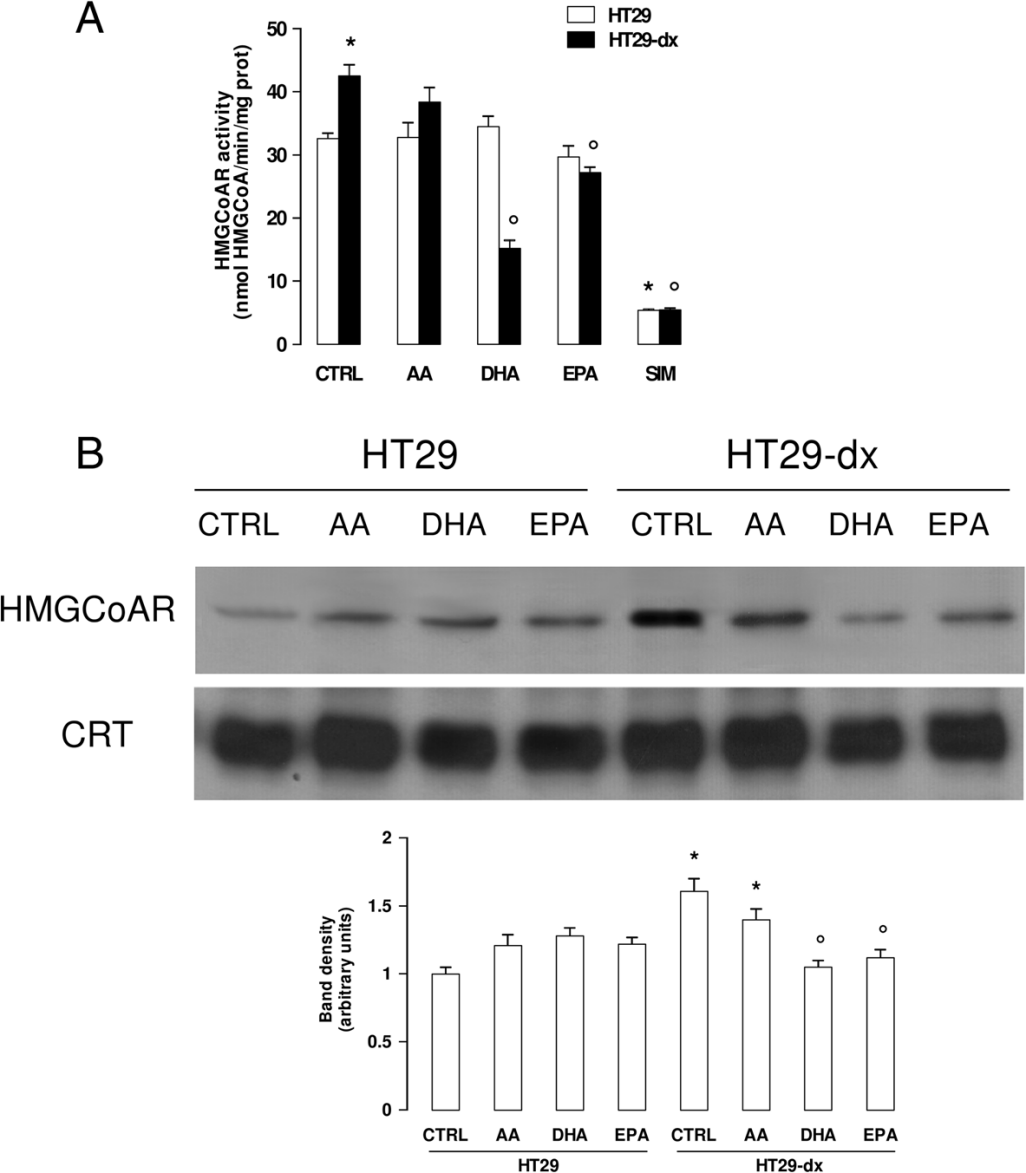
**ω3PUFAs decrease activity and expression of HMGCoAR in chemoresistant colon cancer cells.** HT29 and HT29-dx cells were incubated for 24 h in the absence (CTRL) or in the presence of 50 μM arachidonic acid (AA), docosahexaenoic acid (DHA), eicosapentaenoic acid (EPA). Simvastatin (1 μM for 24 h, SIM) was chosen as HMGCoAR inhibitor. Cells were lysed and centrifuged to collect the microsomal fraction, on which the following investigations were performed. **(A)** HMGCoAR enzymatic activity was measured in duplicate (see Methods). Data are presented as means ± SD (n = 3). Versus CTRL HT29: * p < 0.02; versus CTRL HT29-dx: ° p < 0.05. **(B)** Western blotting experiments were performed using an anti-HMGCoAR antibody; an anti-calreticulin (CRT) antibody was used as a control of equal protein loading. The figure is representative of three experiments with similar results. The band density ratio between HMGCoAR and CRT was expressed as arbitrary units. Versus CTRL HT29: * p < 0.05; versus CTRL HT29-dx: ° p < 0.05.

The higher synthesis of cholesterol in HT29-dx cells was accompanied by higher enzymatic activity (Figure 2A) and protein expression (Figure 2B) of HMGCoAR, compared to HT29 cells. Whereas AA did not modify these parameters, DHA and EPA significantly lowered the activity (Figure 2A) and expression (Figure 2B) of HMGCoAR in chemoresistant cells. Interestingly, they had no effect in chemosensitive cells.

**Figure 3:**
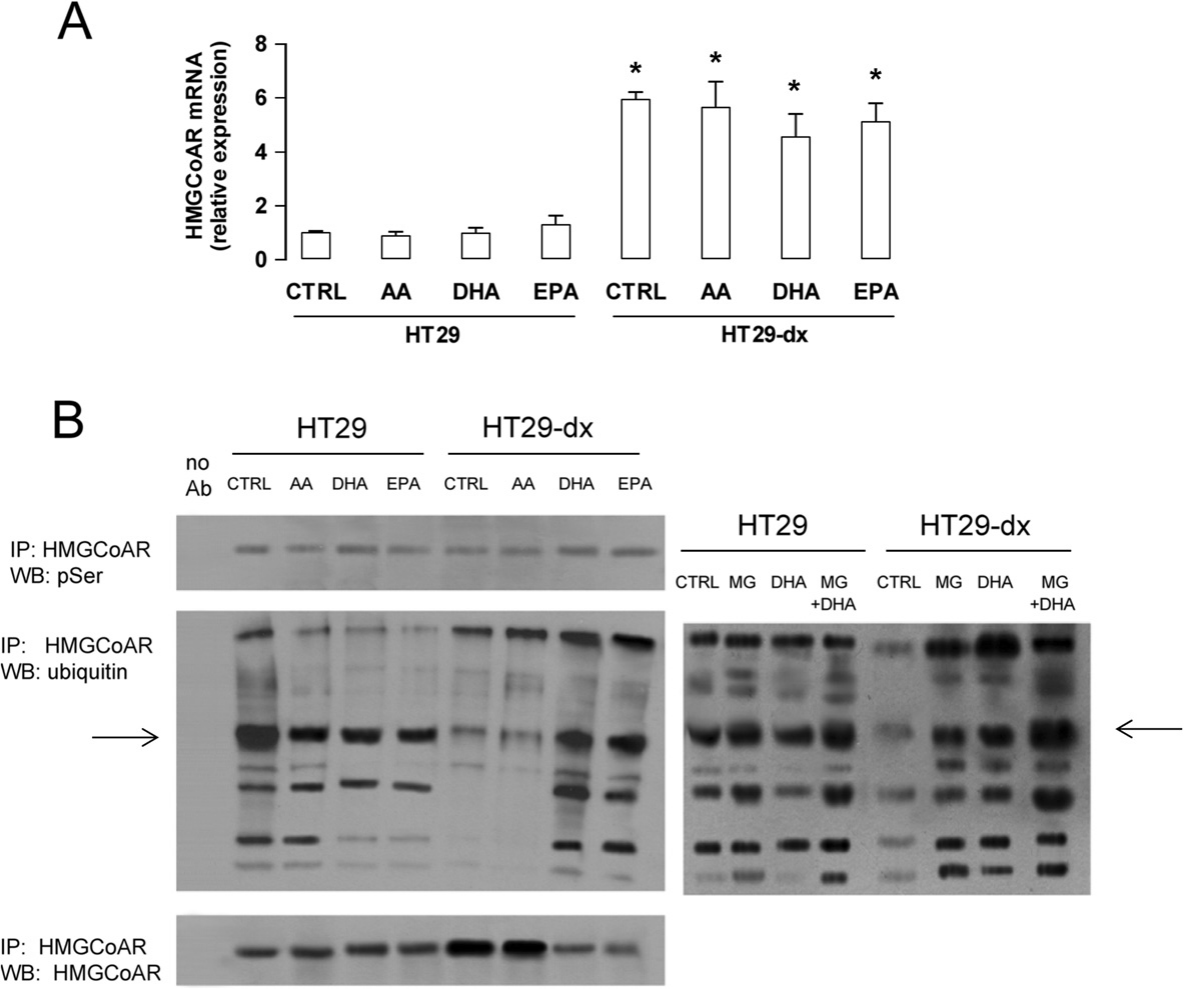
**Effects of ω3PUFAs on HMGCoAR transcription, phosphorylation and ubiquitination in colon cancer cells.** HT29 and HT29-dx cells were incubated for 24 h in the absence (CTRL) or in the presence of 50 μM arachidonic acid (AA), docosahexaenoic acid (DHA), eicosapentaenoic acid (EPA). **(A)** Total RNA was extracted, reverse-transcribed and subjected to qRT-PCR for *HMGCoAR* gene. Measurements were performed in triplicate and data are presented as means ± SD (n = 3). Versus CTRL HT29: * p < 0.005. **(B)** Cells were subjected to ultracentrifugation to isolate the microsomal fraction. Left panel: extracts of microsomal fraction were immunoprecipitated with an anti-HMGCoAR antibody, then probed with anti-phosphoserine (pSer) antibody, anti-ubiquitin antibody or anti-HMGCoAR antibody, to detect the amount of the immunoprecipitated (IP) enzyme. No Ab: extracts in the absence of the anti-HMGCoAR antibody, a condition used as internal control. Right panel: extracts of microsomal fraction were immunoprecipitated with an anti-HMGCoAR antibody, then probed with anti-ubiquitin antibody. As a control of proteasome involvement in HMGCoAR degradation, the proteasome inhibitor MG-132 (10 μM, MG) was added for 16 h, alone or during the last 16 h of the incubation with DHA. The figures are representative of three experiments with similar results. The 95 kDa band corresponding to native HMGCoAR protein is indicated by the arrow.

HT29-dx cells had higher levels of *HMGCoAR* (Figure 3A) and *HMGCoAS* (Additional file 2A) mRNA than HT29 cells, and higher nuclear levels of the transcription factor SREBP-2 (Additional file 2B). Neither ω6PUFA nor ω3PUFAs changed the levels of *HMGCoAR* (Figure 3A) and *HMGCoAS* (Additional file 2A) mRNA, and the amount of nuclear SREBP-2 (Additional file 2B). These results suggest that DHA and EPA did not decrease HMGCoAR expression by down-regulating gene transcription. SREBP-1 was unmodified in each experimental condition and did not differ between HT29 and HT29-dx cells (Additional file 2B).

Since HMGCoAR can be negatively regulated at post-transcriptional level, by phosphorylation on serine [38] or ubiquitination followed by proteasome degradation [39], we next investigated whether ω3PUFAs may affect these events. HMGCoAR was basally phosphorylated on serine in both HT29 and HT29-dx cells without appreciable differences between the two cell populations; PUFAs did not modify the phosphorylation status (Figure 3B, left panel).

In HT29 cells HMGCoAR was highly ubiquitinated, both in the absence or presence of PUFAs. By contrast, the ubiquitination of HMGCoAR was lower in HT29-dx cells; DHA and EPA, but not AA, restored the ubiquitination of HMGCoAR to the same level observed in HT29 cells (Figure 3B, left panel). The proteasome inhibitor MG-132 further increased the amount of ubiquitinated HMGCoAR, in both HT29 and HT29-dx cells, cultured in the absence or presence of DHA (Figure 3B, right panel). These data suggest that the ubiquitination of HMGCoAR was followed by its degradation via the proteasome system.

**Figure 4:**
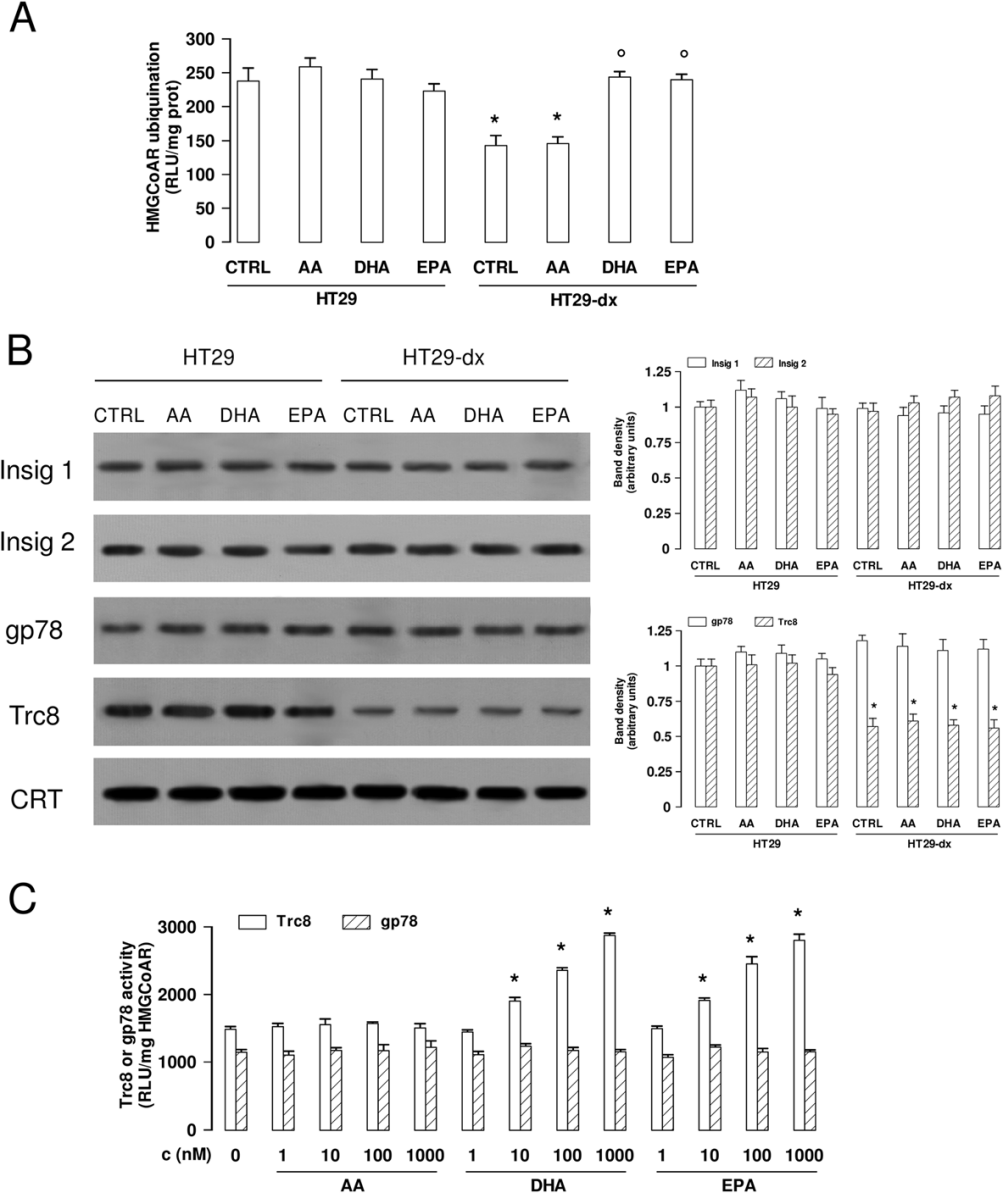
**ω3PUFAs increase the ubiquitination of HMGCoAR by activating the E3 ligase Trc8. (A)** The microsomal fractions from HT29 and HT29-dx cells were incubated for 30 min at 37°, without (CTRL) or with 1 μM arachidonic acid (AA), docosahexaenoic acid (DHA), eicosapentaenoic acid (EPA), in the presence of E1 ubiquitin activating enzyme, E2 ubiquitin conjugating enzyme Ube2g2, ubiquitin and ATP. Samples were immunoprecipitated with an anti-HMGCoAR antibody; ubiquitinated HMGCoAR was measured by a chemiluminescence-based assay. Measurements were performed in triplicate and data are presented as means ± SD (n = 3). Versus CTRL HT29: * p < 0.005; versus CTRL HT29-dx: * p < 0.005. **(B)** HT29 and HT29-dx cells were incubated for 24 h in the absence (CTRL) or presence of 50 μM arachidonic acid (AA), docosahexaenoic acid (DHA) or eicosapentaenoic acid (EPA), then lysed and centrifuged to collect the microsomal fractions. Western blotting experiments were performed using anti-Insig-1, Insig-2, gp78, Trc8 antibodies. The expression of calreticulin (CRT) was measured to check the equal protein loading. The figure is representative of three experiments with similar results. The band density ratio between each protein and CRT was expressed as arbitrary units. Versus CTRL HT29: * p < 0.001. **(C)** Human recombinant HMGCoAR was incubated for 30 min at 37°, without (CTRL) or with various concentrations (1 nM, 10 nM, 100 nM, 1 μM) of arachidonic acid (AA), docosahexaenoic acid (DHA) or eicosapentaenoic acid (EPA), in the presence of E1 ubiquitin activating enzyme, E2 ubiquitin conjugating enzyme Ube2g2, E3 ligase enzymes gp78 or Trc8. The amount of ubiquitinated HMGCoAR was measured by a chemiluminescence-based assay. Measurements were performed in triplicate and data are presented as means ± SD (n = 3). Versus CTRL: * p < 0.05.

The endoplasmic reticulum (ER) proteins Insig-1 and Insig-2 are known to mediate the sterol-dependent degradation of HMGCoAR, by recruiting at least two ER-associated E3 ligases, namely gp78 and Trc8 [39]. gp78 cooperates with the E2 ubiquitin-conjugating enzyme Ube2g2 to ubiquitinate HMGCoAR [40]. All these proteins are components of the so-called ER-associated degradation (ERAD) system, and we investigated whether they are modulated by ω3PUFAs. To measure the activity of the ERAD system in cell extracts, microsomal fractions from HT29 and HT29-dx cells were incubated with an E1 ubiquitin-activating enzyme, Ube2g2 enzyme, ATP and ubiquitin. Then HMGCoAR was isolated by immunoprecipitation and the ubiquitin bound to HMGCoAR was quantified. As shown in Figure 4A, HT29-dx cells exhibited a lower ubiquitination of HMGCoAR, in comparison to HT29 cells. The ubiquitination was not affected by AA. DHA and EPA instead increased the amount of ubiquitinated HMGCoAR in HT29-dx cells to levels comparable to those observed in HT29 cells.

We did not find any difference in the expression of Insig-1, Insig-2 and gp78 between chemosensitive and chemoresistant cells, either untreated or treated with AA, DHA and EPA (Figure 4B). By contrast, Trc8 expression was lower in HT29-dx cells, providing a putative explanation for the lower ubiquitination of HMGCoAR in these cells. Since in HT29-dx cells DHA and EPA did not modify the expression of Trc8 (Figure 4B), but increased the ubiquitination of HMGCoAR versus the control (Figures 3B and 4A), we next investigated whether these PUFAs directly activate Trc8 enzyme. In a cell-free system, containing purified HMGCoAR, recombinant Trc8 and the necessary components of the ubiquitination machinery, DHA and EPA dose-dependently increased the ubiquitination of HMGCoAR, whereas AA was devoid of effects (Figure 4C). On the other hand, DHA and EPA did not increase the ubiquitination when gp78 replaced Trc8 as E3 ligase (Figure 4C), suggesting that they are specific activators of Trc8.

### ω3PUFAs alter the DRMs composition and reduce the DRM-associated Pgp and MRP1 in chemoresistant colon cancer cells

**Figure 5:**
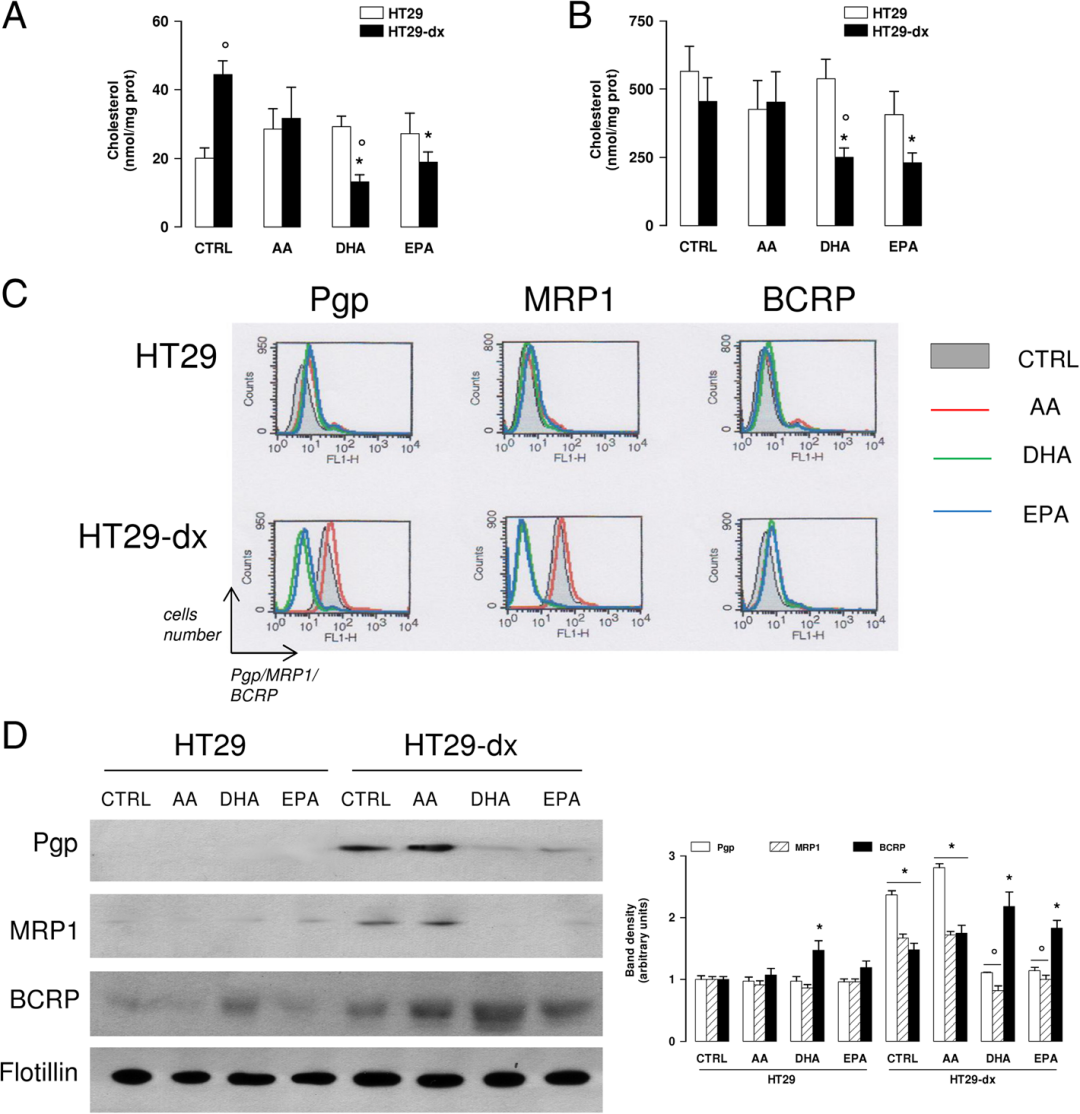
**Effects of ω3PUFAs on cholesterol and ABC transporters in whole cell and DRMs.** HT29 and HT29-dx cells were incubated for 48 h in the absence (CTRL) or in the presence of 50 μM arachidonic acid (AA), docosahexaenoic acid (DHA), eicosapentaenoic acid (EPA). **(A)** Total lipids were extracted and the amount of cholesterol was measured in triplicate as reported under Methods. Data are presented as mean ± SD (n = 3). Versus respective CTRL: * p < 0.01; HT29-dx versus HT29 cells: ° p < 0.01. **(B)** DRMs were isolated as detergent-resistant membranes by separation on sucrose gradient; then the amount of cholesterol was measured as reported in Methods. Data are presented as mean ± SD (n = 4). Versus respective CTRL: * p < 0.05; HT29-dx versus HT29 cells: ° p < 0.01. **(C)** Surface levels of Pgp, MRP1 and BCRP were measured in non-permeabilized cells by flow cytometry. The figures shown here are representative of three similar experiments, performed in triplicate. **(D)** Western blot detection of Pgp, MRP1 and BCRP on DRM extracts. Flotillin expression was used as control of equal protein loading. The figure is representative of two experiments with similar results. The band density ratio between each protein and flotillin was expressed as arbitrary units. Versus CTRL HT29: * p < 0.05; versus CTRL HT29-dx: p < 0.005.

Consistently with the higher rate of cholesterol synthesis (Figure 1A), HT29-dx cells had twice more cholesterol in whole cell than HT29 cells (Figure 5A). DHA and EPA decreased cholesterol levels only in HT29-dx cells (Figure 5A). As expected, DRMs were characterized by a high cholesterol content in both cell lines (Figure 5B); again, only in HT29-dx cells DHA and EPA were able to significantly decrease cholesterol (Figure 5B).

PUFAs were incorporated both in whole cell lipids (Tables 1 and 2) and DRMs (Tables 3 and 4). Compared to the other PUFAs, EPA was integrated with higher efficiency in whole cell lipids in HT29 cells (11.21-fold increase) and further metabolized to docosapentaenoic acid (DPA); it was incorporated with a bit lower efficiency in HT29-dx cells (7.03-fold increase) without any further metabolism (Tables 1 and 2). DHA showed the same integration efficiency in both cell lines (6.63-fold increase in HT29 cells and 5.86-fold increase in HT29-dx cells). ω3PUFAs significantly reduced the AA whole content in both cell lines.

**Table 1:** Fatty acids composition of whole HT29 cells

	**CTRL**	**AA**	**DHA**	**EPA**
**C16:0**	30.131 ± 2.328	28.882 ± 2.166	30.803 ± 2.728	29.952 ± 3.450
**C16:1**	10.990 ± 1.086	8.669 ± 0.877	9.091 ± 1.042	7.308 ± 1.693
**C18:0**	13.155 ± 0.842	12.327 ± 0.595	12.052 ± 1.093	13.176 ± 1.604
**C18:1**	29.677 ± 1.260	30.412 ± 1.259	29.317 ± 2.910	26.824 ± 2.418
**C18:2**	4.003 ± 0.547	2.753 ± 0.221	2.612 ± 0.322	2.681 ± 0.317
**C18:3 ω-3**	0.513 ± 0.143	1.125 ± 0.202*	0.565 ± 0.073	0.723 ± 0.112
**C18:3 ω-6**	0.416 ± 0.060	0.129 ± 0.020*	0.176 ± 0.038*	0.131 ± 0.240
**C20:3**	1.222 ± 0.176	0.814 ± 0.035	0.833 ± 0.060	0.791 ± 0.031
**C20:4 (AA)**	6.032 ± 1.164	11.394 ± 1.177**	3.687 ± 0.332*	3.594 ± 0.344*
**C20:5 (EPA)**	1.536 ± 0.337	1.071 ± 0.220	1.638 ± 0.097	11.570 ± 2.685**
**C22:5**	0.743 ± 0.126	0.706 ± 0.123	0.886 ± 0.101	1.561 ± 0.306**
**C22:6 (DHA)**	1.760 ± 0.380	1.716 ± 0.350	8.339 ± 2.375**	1.689 ± 0.300
**SFA**^**a**^	43.285 ± 2.860	41.209 ± 2.345	42.855 ± 3.196	43.128 ± 4.836
**MUFA**^**b**^	40.667 ± 1.579	39.081 ± 1.561	38.409 ± 3.640	34.132 ± 3.632
**ω-6**	8.990 ± 0.759	15.091 ± 1.196**	7.308 ± 0.629	7.197 ± 0.608
**ω-3**	4.553 ± 0.738	4.620 ± 0.643	11.429 ± 2.477**	15.543 ± 3.073**
**ω-6/ω-3**	2.386 ± 0.307	3.371 ± 0.295	0.748 ± 0.172**	0.513 ± 0.095**
**Fold increase**^**c**^		3.008	6.634	11.209

HT29 cells were left untreated (CTRL) or treated with AA, DHA, EPA (50 μM for 48 h).

^a^SFA: saturated fatty acids; ^b^MUFA: mono-unsaturated fatty acids.

^c^Fold increase: AA, DHA or EPA in treated cells versus AA, DHA or EPA in untreated cells, respectively.

Data are presented as percentage of total fatty acids, mean ± SE (n = 3). Significance versus CTRL: *p < 0.05; **p < 0.01.

**Table 2:** Fatty acids composition of whole HT29-dx cells

	**CTRL**	**AA**	**DHA**	**EPA**
**C16:0**	28.821 ± 2.333	28.352 ± 2.367	30.020 ± 3.349	29.630 ± 2.872
**C16:1**	10.532 ± 0.945	9.955 ± 1.003	9.027 ± 0.986	9.652 ± 0.905
**C18:0**	12.386 ± 1.082	11.401 ± 1.731	12.870 ± 2.455	12.229 ± 1.465
**C18:1**	30.878 ± 1.510	31.793 ± 2.214	30.288 ± 3.019	31.674 ± 2.552
**C18:2**	4.266 ± 0.792	2.563 ± 0.239	2.182 ± 0.368	3.025 ± 0.184
**C18:3 ω-3**	0.438 ± 0.068	0.955 ± 0.165**	0.514 ± 0.094	0.659 ± 0.036*
**C18:3 ω-6**	**0.113 ± 0.011§**	0.111 ± 0.013	0.147 ± 0.038	0.107 ± 0.009
**C20:3**	1.181 ± 0.209	0.828 ± 0.027	0.874 ± 0.107	0.895 ± 0.080
**C20:4 (AA)**	6.571 ± 1.563	9.813 ± 0.560*	3.182 ± 0.590*	3.694 ± 0.431*
**C20:5 (EPA)**	1.707 ± 0.425	0.812 ± 0.060	1.477 ± 0.227	5.852 ± 0.677**
**C22:5**	0.875 ± 0.080	0.823 ± 0.102	0.835 ± 0.166	1.101 ± 0.106
**C22:6 (DHA)**	2.280 ± 0.467	1.594 ± 0.274	8.345 ± 2.915*	1.481 ± 0.350
**SFA**^**a**^	41.208 ± 2.757	40.753 ± 3.507	42.890 ± 5.505	41.860 ± 4.067
**MUFA**^**b**^	41.410 ± 2.336	41.748 ± 3.196	39.555 ± 3.886	41.326 ± 3.443
**ω-6**	12.083 ± 2.310	13.315 ± 0.539	6.385 ± 0.735*	7.721 ± 0.309*
**ω-3**	5.300 ± 0.904	4.184 ± 0.315	11.171 ± 2.988*	9.094 ± 0.697**
**ω-6/ω-3**	2.271 ± 0.198	3.208 ± 0.129**	0.679 ± 0.162**	0.863 ± 0.070**§
**Fold increase**^**c**^		2.782	5.862	7.028

HT29-dx cells were left untreated (CTRL) or treated with AA, DHA, EPA (50 μM for 48 h).

^a^SFA: saturated fatty acids; ^b^MUFA: mono-unsaturated fatty acids.

^c^Fold increase: AA, DHA or EPA in treated cells versus AA, DHA or EPA in untreated cells, respectively.

Data are presented as percentage of total fatty acids, mean ± SE (n = 3). Significance versus CTRL: *p < 0.05; **p < 0.01; versus the corresponding treatment in HT29 cells: **§**p < 0.05.

**Table 3:** Detergent resistant membrane (DRM) fatty acids composition of HT29 cells

	**CTRL**	**AA**	**DHA**	**EPA**
**C16:0**	35.973 ± 2.332	31.712 ± 1.344	31.684 ± 1.527	31.238 ± 2.09
**C16:1**	6.470 ± 1.522	8.098 ± 0.457	6.761 ± 1.409	9.479 ± 0.789
**C18:0**	14.923 ± 0.789	15.291 ± 0.352	16.787 ± 1.179	14.685 ± 0.971
**C18:1**	32.203 ± 1.068	30.241 ± 2.900	32.918 ± 2.472	30.065 ± 2.061
**C18:2**	3.184 ± 0.283	3.252 ± 0.895	2.871 ± 0.281	4.048 ± 1.115
**C18:3 ω-3**	0.352 ± 0.035	0.610 ± 0.150	0.389 ± 0.015	0.322 ± 0.084
**C18:3 ω-6**	0.521 ± 0.154	0.326 ± 0.059	0.389 ± 0.138	2.567 ± 1.893
**C20:3**	0.835 ± 0.160	0.308 ± 0.018	0.690 ± 0.022	0.581 ± 0.085
**C20:4 (AA)**	3.183 ± 0.477	8.455 ± 1.782**	3.303 ± 0.159	3.466 ± 0.428
**C20:5 (EPA)**	0.888 ± 0.178	0.589 ± 0.036	1.260 ± 0.353	1.827 ± 0.304*
**C22:5**	0.394 ± 0.066	0.590 ± 0.133	0.388 ± 0.101	0.565 ± 0.117
**C22:6 (DHA)**	1.077 ± 0.157	1.069 ± 0.082	2.553 ± 0.494**	1.158 ± 0.178
**SFA**^**a**^	50.895 ± 2.810	47.003 ± 1.387	48.471 ± 1.743	45.923 ± 2.935
**MUFA**^**b**^	38.673 ± 2.231	38.339 ± 3.201	39.679 ± 2.388	39.544 ± 2.518
**ω-6**	7.202 ± 0.862	12.650 ± 2.766*	6.863 ± 0.338	8.095 ± 1.397
**ω-3**	2.708 ± 0.311	2.711 ± 0.193	4.591 ± 0.732*	3.872 ± 0.358*
**ω-6/ω-3**	2.735 ± 0.234	4.576 ± 0.579**	1.577 ± 0.183**	2.122 ± 0.374
**Fold increase**^**c**^		2.403	2.116	2.199

HT29 cells were left untreated (CTRL) or treated with AA, DHA, EPA (50 μM for 48 h).

^a^SFA: saturated fatty acids; ^b^MUFA: mono-unsaturated fatty acids.

^c^Fold increase: AA, DHA or EPA in treated cells versus AA, DHA or EPA in untreated cells, respectively.

Data are presented as percentage of total fatty acids, mean ± SE (n = 4). Significance versus CTRL: *p < 0.05; **p < 0.01.

**Table 4:** Detergent resistant membrane (DRM) fatty acids composition of HT29-dx cells

	**CTRL**	**AA**	**DHA**	**EPA**
**C16:0**	33.091 ± 2.128	31.588 ± 1.305	32.446 ± 2.793	35.242 ± 1.836
**C16:1**	9.598 ± 0.716	9.155 ± 0.742	7.628 ± 2.111	9.443 ± 1.181
**C18:0**	14.605 ± 0.786	14.957 ± 0.709	16.538 ± 1.964	17.281 ± 0.933
**C18:1**	31.885 ± 1.922	30.603 ± 1.864	29.766 ± 2.366	30.369 ± 2.772
**C18:2**	3.511 ± 0.512	3.059 ± 0.934	3.107 ± 0.770	2.950 ± 0.340
**C18:3 ω-3**	0.351 ± 0.028	0.630 ± 0.052*	**0.460 ± 0.011§§**	0.480 ± 0.057
**C18:3 ω-6**	0.480 ± 0.160	0.540 ± 0.183	0.455 ± 0.156	0.435 ± 0.226
**C20:3**	0.876 ± 0.059	0.700 ± 0.055	0.692 ± 0.032	0.654 ± 0.059
**C20:4 (AA)**	2.978 ± 0.266	7.599 ± 0.421**	3.093 ± 0.403	2.829 ± 0.395
**C20:5 (EPA)**	0.711 ± 0.053	0.717 ± 0.396	1.113 ± 0.214	3.498 ± 1.205**
**C22:5**	0.595 ± 0.141	0.507 ± 0.089	1.011 ± 0.619	0.481 ± 0.201
**C22:6 (DHA)**	1.320 ± 0.113	1.365 ± 0.113	4.500 ± 1.286**	1.258 ± 0.104
**SFA**^**a**^	47.696 ± 2.853	46.545 ± 1.903	48.984 ± 4.545	48.203 ± 5.614
**MUFA**^**b**^	41.483 ± 2.452	39.759 ± 2.546	37.394 ± 3.993	39.812 ± 3.942
**ω-6**	7.364 ± 0.609	10.403 ± 0.498*	6.891 ± 0.643	6.433 ± 0.401
**ω-3**	2.977 ± 0.266	3.218 ± 0.270	7.490 ± 1.974*	5.878 ± 1.461*
**ω-6/ω-3**	2.680 ± 0.160	3.141 ± 0.309	1.055 ± 0.287*	1.312 ± 0.378
**Fold increase**^**c**^		2.758	3.024	4.821

HT29-dx cells were left untreated (CTRL) or treated with AA, DHA, EPA (50 μM for 48 h).

^a^SFA: saturated fatty acids; ^b^MUFA: mono-unsaturated fatty acids.

^c^Fold increase: AA, DHA or EPA in treated cells versus AA, DHA or EPA in untreated cells, respectively.

Data are presented as percentage of total fatty acids, mean ± SE (n = 4). Significance versus CTRL: *p < 0.05; **p < 0.01; versus the corresponding treatment in HT29 cells: §§ p < 0.01.

Both DHA and EPA were integrated in DRM phospholipids even if with lower efficiency compared to their incorporation in whole cell lipids (Tables 3 and 4); in particular in HT29-dx cells they were respectively 4.50 ± 1.29% and 3.50 ± 1.20% of total fatty acids (Table 4).

HT29-dx cells had higher amounts of Pgp and MRP1 on their surface (Figure 5C and Table 5). Interestingly, the decrease in cell membrane cholesterol elicited by DHA and EPA was accompanied by diminished surface levels of Pgp and MRP1, which became similar to the levels detected in HT29 cells (Figure 5C and Table 5). In particular, Pgp and MRP1 were more expressed in DRMs of HT29-dx cells (Figure 5D) and their DRM-association was significantly reduced by ω3PUFAs (Figure 5D). AA, which was incorporated in whole cell lipids (Tables 1 and 2) and DRMs (Tables 3 and 4), without changing the amount of cholesterol (Figure 5A-B), did not modify Pgp and MRP1 levels (Figure 5C-D; Table 5).

**Table 5:** Median and mean fluorescence intensity (MFI) of surface Pgp, MRP1 and BCRP in HT29 and HT29-dx cells

	**Pgp**	**Pgp**	**MRP1**	**MRP1**	**BCRP**	**BCRP**
**Sample**	**Median**	**MFI**	**Median**	**MFI**	**Median**	**MFI**
HT29 CTRL	8.24 ± 0.59	7.21 ± 0.11	5.15 ± 0.43	4.61 ± 0.41	5.37 ± 0.25	4.71 ± 0.26
HT29 AA	10.02 ± 2.25	8.58 ± 1.11	6.35 ± 0.54	5.52 ± 0.36	6.80 ± 0.21	5.62 ± 0.37
HT29 DHA	14.93 ± 2.02	8.74 ± 0.92	5.49 ± 0.29	5.25 ± 0.44	7.12 ± 0.47	6.49 ± 0.36
HT29 EPA	11.73 ± 1.01	10.09 ± 0.98	6.00 ± 0.47	6.55 ± 0.34	6.15 ± 0.29	5.57 ± 0.19
HT29-dx CTRL	34.84 ± 3.66*	31.06 ± 3.52*	34.01 ± 2.15*	32.20 ± 1.08*	5.15 ± 0.23	4.96 ± 0.23
HT29-dx AA	48.83 ± 4.72*	43.32 ± 4.98*	44.04 ± 5.53*	41.05 ± 5.11*	7.74 ± 0.83	7.23 ± 0.41
HT29-dx DHA	6.41 ± 0.52°	5.83 ± 0.41°	3.38 ± 0.41°	3.16 ± 0.37°	7.69 ± 0.79	7.23 ± 0.59
HT29-dx EPA	8.26 ± 0.51°	7.50 ± 0.21**°**	3.31 ± 0.24°	2.89 ± 0.13°	8.17 ± 0.89	7.64 ± 0.48

HT29 and HT29-dx cells were left untreated (CTRL) or treated with AA, DHA, EPA (50 μM for 48 h).

Data were calculated with the Cell Quest software and are presented as mean ± SD (n = 3). Versus CTRL HT29: *p < 0.002; versus CTRL HT29-dx: ° p < 0.001.

The surface level of BCRP was similar in HT29 and HT29-dx cells (Figure 5C and Table 5), but the DRM-associated BCRP was slightly higher in HT29-dx cells (Figure 5D). According to the densitometric analysis, DHA and – at lesser extent – AA and EPA increased the amount of DRM-associated BCRP in both HT29 and HT29-dx cells (Figure 5D). The changes of ABC transporters on cell surface were not due to differences in the absolute amount of Pgp, MRP1 and BCRP: all these proteins were higher in the whole cell lysate of HT29-dx cells compared to HT29 cells and were not affected by AA, DHA or EPA (Additional file 3). These results suggest that the changes of Pgp and MRP1 surface levels are likely due to the redistribution between plasma membrane and cytosol.

### ω3PUFAs sensitize multidrug-resistant colon cancer cells to the antitumor effects of Pgp and MRP1 substrates

**Figure 6:**
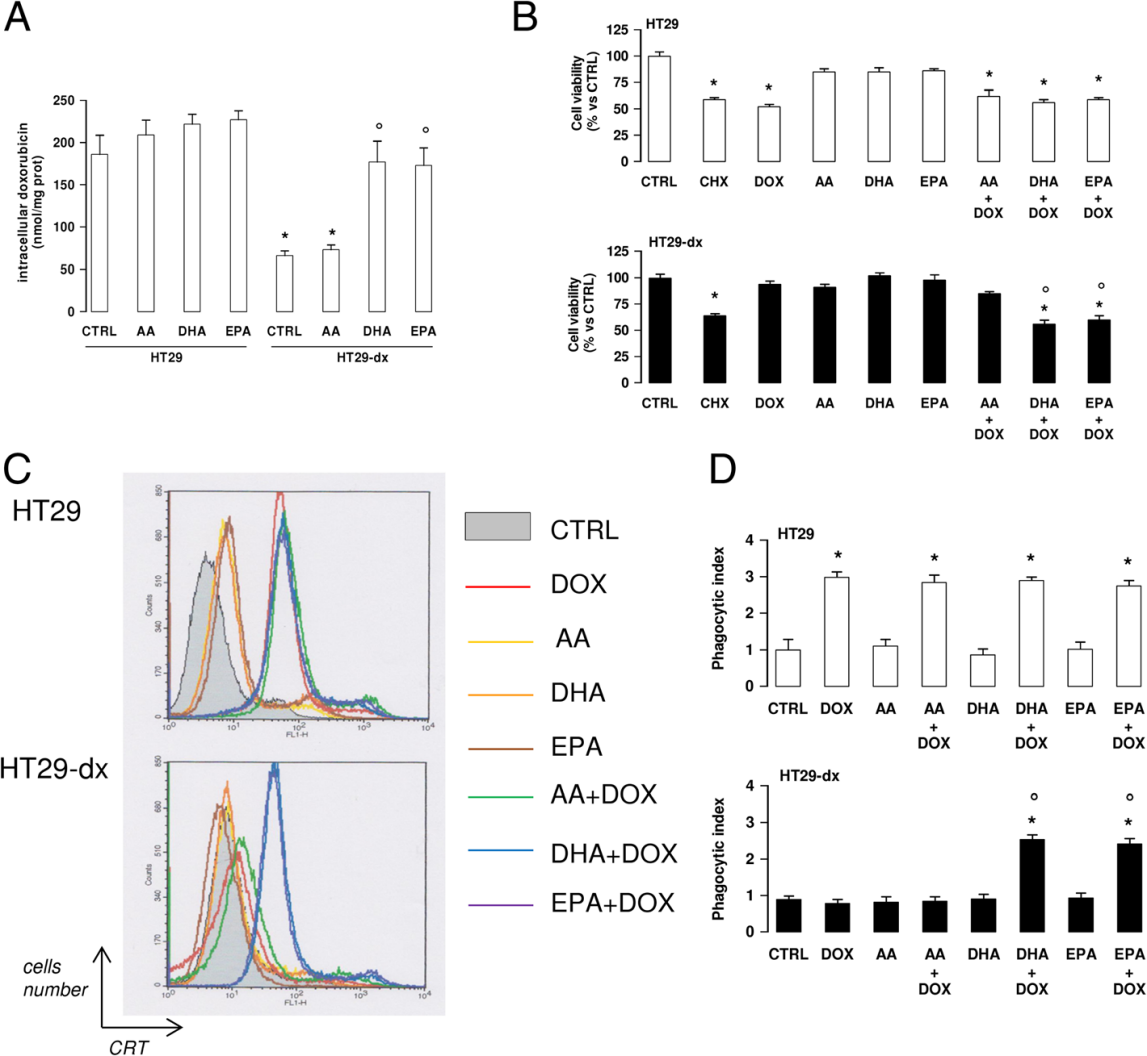
**ω3PUFAs restore doxorubicin cytotoxicity in chemoresistant colon cancer cells.** HT29 and HT29-dx cells were incubated for 48 h in the absence (CTRL) or in the presence of 50 μM arachidonic acid (AA), docosahexaenoic acid (DHA), eicosapentaenoic acid (EPA). 5 μM doxorubicin (DOX) was added for 24 h, alone or during the last 24 h of incubation with fatty acids. Cycloheximide (4 μM for 24 h, CHX) was chosen as positive control of cytotoxicity in both chemosensitive and chemoresistant cells. **(A)** The intracellular accumulation of doxorubicin was measured fluorimetrically in duplicate. Data are presented as means ± SD (n = 3). Versus CTRL HT29: * p < 0.005; versus CTRL HT29-dx: ° p < 0.005. **(B)** Cells were stained with Neutral Red solution and the absorbance of viable cells was measured in triplicate spectrophotometrically. Data are presented as means ± SD (n = 4). Versus respective CTRL: * p < 0.002; versus DOX alone: ° p < 0.005. **(C)** Surface levels of calreticulin were measured in non-permeabilized cells by flow cytometry. The figures shown here are representative of three similar experiments, performed in triplicate. **(D)** The phagocytosis rate by DCs was evaluated in duplicate by flow cytometry. Data are presented as means ± SD (n = 4). Versus respective CTRL: * p < 0.05; versus DOX alone: p < 0.005.

The anthracycline doxorubicin, used to generate the resistant HT29-dx cell population [41], is a substrate of both Pgp and MRP1 [27]. Although anthracyclines are not used in colon cancer therapy, we chose doxorubicin as a reliable tool to clarify whether ω3PUFAs chemosensitize resistant cells to anticancer drugs effluxed by Pgp and MRP1. Doxorubicin was indeed less accumulated in HT29-dx cells (Figure 6A) and did not reduce their viability (Figure 6B). In correlation with the reduction of Pgp and MRP1 at cell surface, DHA and EPA increased the intracellular doxorubicin retention (Figure 6A) and restored its cytotoxicity in HT29-dx cells (Figure 6B). Anthracyclines are amongst the few anticancer drugs able to kill cancer cells by exerting direct cytotoxicity on tumor cell and by inducing an immunogenic cell death. This type of cell death is characterized by the extracellular release of ATP and HMGB1, by the surface translocation of calreticulin, and by the subsequent phagocytosis of tumor cells by dendritic cells (DCs) [42,43]. While doxorubicin elicited all these events in HT29 cells, it was ineffective in HT29-dx cells (Additional file 4A-B; Figure 6C-D; Table 6). EPA and DHA, which did not further enhance the effects of doxorubicin in chemosensitive cells, restored the doxorubicin-mediated release of extracellular ATP (Additional file 4A) and HMGB1 (Additional file 4B), the translocation of calreticulin on cell surface (Figure 6C; Table 6) and the DC-mediated phagocytosis of HT29-dx cells (Figure 6D), confirming that they fully restored the pro-immunogenic effects of doxorubicin. Of note, neither DHA nor EPA alone increased ATP and HMGB1 release, calreticulin translocation and cells phagocytosis (Additional file 4A-B; Figure 6C-D; Table 6), suggesting that they were not immune-activating agents *per se*, but only enhancers of doxorubicin activity. Again, AA was completely ineffective in restoring doxorubicin cytotoxicity and pro-immunogenic effects in HT29-dx cells (Additional file 4; Figure 6; Table 6).

**Table 6:** Median and mean fluorescence intensity (MFI) of surface calreticulin (CRT) in HT29 and HT29-dx cells

	**CRT**	**CRT**
**Sample**	**Median**	**MFI**
HT29 CTRL	4.96 ± 0.17	4.29 ± 0.32
HT29 DOX	57.59 ± 4.87*	53.28 ± 6.25*
HT29 AA	8.50 ± 1.69	7.37 ± 0.96
HT29 DHA	9.51 ± 2.14	7.57 ± 0.84
HT29 EPA	10.84 ± 2.39	8.74 ± 1.28
HT29 AA + DOX	65.44 ± 4.91*	67.17 ± 6.32*
HT29 DHA + DOX	70.40 ± 4.85*	58.82 ± 4.82*
HT29 EPA + DOX	68.16 ± 5.13*	59.35 ± 3.31*
HT29-dx CTRL	8.99 ± 1.89	8.51 ± 0.27
HT29-dx DOX	10.08 ± 2.78	10.09 ± 0.31
HT29-dx AA	11.41 ± 2.49	9.06 ± 0.97
HT29-dx DHA	10.85 ± 2.11	8.66 ± 0.41
HT29-dx EPA	6.77 ± 0.82	6.55 ± 0.53
HT29-dx AA + DOX	17.68 ± 5.15	14.72 ± 4.66
HT29-dx DHA + DOX	56.74 ± 4.60*°	46.56 ± 2.64*°
HT29-dx EPA + DOX	56.25 ± 5.21*°	44.11 ± 3.61*°

HT29 and HT29-dx cells were left untreated (CTRL) or treated with AA, DHA, EPA (50 μM for 48 h).

Data were calculated with the Cell Quest software and are presented as mean ± SD (n = 3). Versus CTRL HT29: *p < 0.001; HT29-dx DHA + DOX/EPA + DOX versus HT29-dx DOX: ° p < 0.002.

**Figure 7:**
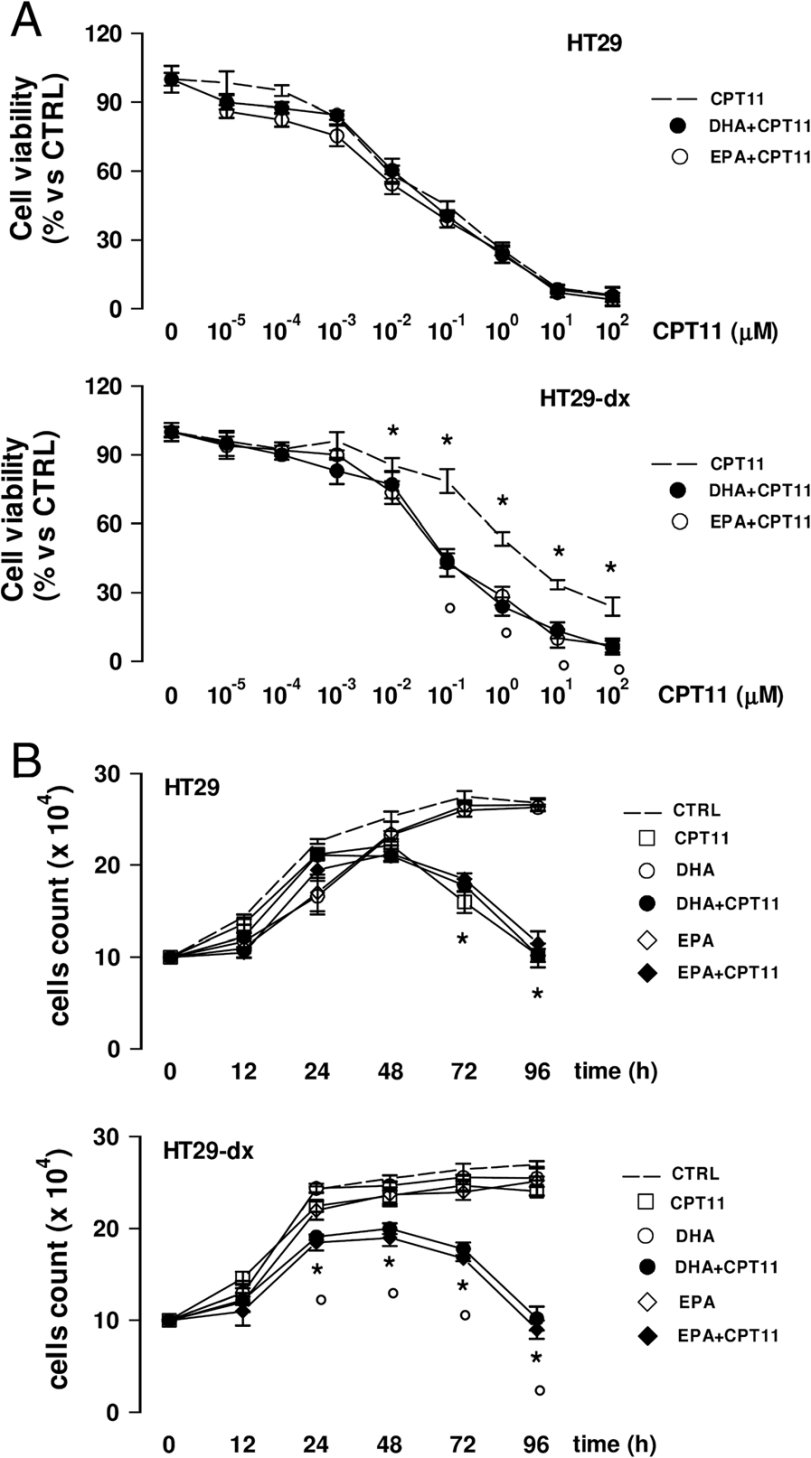
**ω3PUFAs restore the anti-proliferative effects of irinotecan in chemoresistant colon cancer cells.** HT29 and HT29-dx cells were incubated for 48 h in the absence (CTRL) or presence of 50 μM docosahexaenoic acid (DHA) or eicosapentaenoic acid (EPA). **(A)** Irinotecan (CPT11) was added at increasing concentrations in the last 24 h. Cells were stained with Neutral Red solution and the absorbance of viable cells was measured in triplicate spectrophotometrically. Data are presented as means ± SD (n = 4). Versus respective CTRL: * p < 0.001; versus CPT11 alone: ° p < 0.005. **(B)** 10,000 HT29 and HT29-dx cells were seeded in 96-wells plates. After 8 h, the medium was replaced with fresh medium (CTRL), medium containing 50 μM docosahexaenoic acid (DHA), 50 μM eicosapentaenoic acid (EPA) or 1 μM irinotecan (CPT11), alone or in combination. Cell proliferation was monitored at 12, 24, 48, 72, 96 h by crystal violet staining. Measurements were performed in triplicate and data are presented as means ± SD (n = 4). Versus respective CTRL: * p < 0.01; versus CPT11 alone: ° p < 0.01.

The rescue of chemosensitivity was not limited to doxorubicin: indeed, the IC50 of irinotecan, another substrate of Pgp [44], was higher in HT29-dx cells than in HT29 cells (Figure 7A). The drug effectively reduced the proliferation of HT29 cells, not of HT29-dx cells (Figure 7B). DHA and EPA lowered the IC50 of irinotecan in HT29-dx cells to a value similar to HT29 cells (Figure 7A). Moreover, they restored the antiproliferative effect of irinotecan in drug-resistant cells (Figure 7B).

## Discussion

In this work we investigated whether ω3PUFAs – DHA and EPA – chemosensitize MDR colon cancer cells, by modulating the endogenous synthesis of cholesterol and the cholesterol amount in plasma membrane, two factors that affect ABC transporters activity and determine a MDR phenotype [25,26,28,30].

PUFAs have been reported to induce apoptosis in HT29 cells at concentrations higher than 200 μM after 24 h [15]. They are cytotoxic in colon cancer cells at 50 μM for prolonged periods (e.g. 6 days) [45], but do not reduce cell survival after 72 h [46]. Since we used ω3PUFAs and ω6PUFA at 50 μM for 24 h, it is likely that we did not detect any toxicity because of the short incubation time. Interestingly, chemosensitive and chemoresistant cells displayed equal sensitivity to the toxic effects of the highest concentrations of PUFAs, suggesting that the MDR phenotype is not important for the PUFAs’ cytotoxicity. The similar toxicity profile observed in HT29 and HT29-dx cells might also suggest that PUFAs are not effluxed by the ABC transporters present in HT29-dx cells.

Within cells, protein-bound fatty acids may reach concentrations up to 100 μM [15,47]. In order to work at a non-toxic and physiological-like concentration of PUFAs, we used ω3PUFAs and ω6PUFA at 50 μM in all the functional assays.

AA significantly reduced the *de novo* synthesis of cholesterol only at a cytotoxic concentration (200 μM), suggesting that such decrease was a non-specific effect. 50 μM DHA and EPA did not reduce the cholesterol synthesis in chemosensitive HT29 cells, but significantly decreased it in chemoresistant HT29-dx cells. Since the basal rate of cholesterol synthesis was higher in HT29-dx cells than in HT29 cells, it is not surprising that the effect of cholesterol-lowering agents – such as DHA and EPA – was more evident in MDR cells. On the other hand, the different effects exerted by ω3PUFAs in chemosensitive and chemoresistant cells may be indicative of different regulation mechanisms in cholesterol synthesis. HT29-dx cells had higher activity and expression of HMGCoAR mRNA and protein, accompanied by higher nuclear levels of the transcriptional activator SREBP-2. SREBP-2 is up-regulated by intracellular sterols and by the transcription factor hypoxia inducible factor-1α (HIF-1α) [48], which is constitutively active in HT29-dx cells also under normoxic conditions, but undetectable in HT29 cells [26]. The increased activation of HIF-1α/SREBP-2 axis may explain why HT29-dx cells have higher levels of *HMGCoAR* and *HMGCoAS* mRNA, despite the higher levels of cholesterol [25,26]. It is unlikely however that ω3PUFAs interfere with this axis in HT29-dx cells: they indeed reduced HMGCoAR activity and protein in chemoresistant cells, but did not decrease the nuclear translocation of SREBP-2 and the transcription of *HMGCoAR* gene. Our data are partially in contrast with previous works showing that DHA activated SREBP-2 in SW620 colon cancer cells [23,24], without changing however the transcription of *HMGCoAR*[23]. As far as we know, these works were performed on colon cancer cells without a MDR phenotype; this may explain the different behavior of DHA in SW620 cells and in chemoresistant HT29-dx cells. Also in our hands DHA and EPA did not produce any changes in chemosensitive HT29 cells, which maintained lower HMGCoAR activity and expression, and lower nuclear SREBP-2 compared with HT29-dx cells.

Since DHA and EPA only reduced the amount of HMGCoAR protein, without changing the *HMGCoAR* mRNA or the enzyme phosphorylation, we next wondered whether ω3PUFAs modulated HMGCoAR degradation via the ubiquitin/proteasome system. Interestingly, MDR cells showed a lower level of enzyme ubiquitination than chemosensitive cells, a situation that may explain the increased basal amount of HMGCoAR protein. In both cell lines, the proteasome inhibitor MG-132 further increased the amount of ubiquitinated HMGCoAR, suggesting that the ubiquitination was followed by proteasomal degradation. Of note, whereas AA did not affect the rate of ubiquitination, DHA and EPA increased the ubiquitination of HMGCoAR in HT29-dx cells but not in HT29 cells.

When we analyzed the ubiquitination of HMGCoAR in microsomal fraction, we found that the activity of the ERAD system was significantly lower in MDR cells; DHA and EPA increased ERAD activity to the same levels of HT29 cells. In order to investigate the putative target of ω3PUFAs, we first analyzed the expression of Insig-1 and -2, which cooperate with the ER-associated ubiquitin E3 ligases gp78 and Trc8 in the degradation of HMGCoAR [39]. We did not detect any difference between HT29 and HT29-dx cells, in the absence or presence of AA, DHA and EPA, except for Trc8. This E3 ligase was more expressed in HT29 cells than in HT29-dx cells: such difference may explain the differential expression and degradation of HMGCoAR between chemosensitive and chemoresistant cells. Since neither DHA nor EPA changed the expression of Trc8 and gp78, but they both increased the ubiquitination of HMGCoAR, we hypothesized that they were direct activators of Trc8 and/or gp78. To confirm this hypothesis, we set up a cell-free system, containing the human recombinant HMGCoAR protein, the E2 ubiquitin conjugating enzyme Ube2g2, which mediates the degradation of several ER-associated proteins [49] including HMGCoAR [40], and the recombinant gp78 or Trc8 proteins. DHA and EPA dose-dependently increased the ubiquitination of HMGCoAR by activating the Ube2g2-Trc8 system. Since Trc8 was more expressed in HT29 cells, where DHA and EPA did not increase the ubiquitination of HMGCoAR, we may speculate that Trc8 works at its maximal rate in chemosensitive cells and cannot be further activated by ω3PUFAs. By contrast, in HT29-dx cells, where the amount of Trc8 was lower, the activating effect of DHA and EPA became more evident. To our knowledge, this is the first work reporting that ω3PUFAs activate the E3 ligase Trc8. This observation also provides mechanistic insights to explain previous works, reporting that DHA increases the activity of ERAD system [23,24], and the proteasomal degradation of specific proteins in colon cancer [50]. Contrarily to DHA and EPA, AA had no effect on Trc8, in keeping with its inefficacy in reducing HMGCoAR and cholesterol synthesis. ω3PUFAs and ω6PUFAs have different conformation and steric hindrance; these factors can determine the selective interactions with target proteins and the specificity of their effects.

A striking consequence of down-regulating cholesterol synthesis in HT29-dx cells is the reduction of the cholesterol amount in DRMs, which we extracted as described [51,52]. It has been reported that DRMs contain about 40% of Pgp in drug-resistant HT29 cells [53]. Pgp, MRP1 and BCRP associated with DRMs are active and the peculiar physicochemical properties of DRM domains – such as the presence of cholesterol – preserve the transporters activity [29,30,53,54]. The depletion of cholesterol from DRMs indeed induces the shift of Pgp into soluble cell fractions and the decrease of its activity [54]. This observation is in keeping with the decrease of surface- and DRM-associated Pgp that we observed in HT29-dx cells treated with EPA and DHA. The decrease of surface Pgp was not accompanied by a reduced amount of Pgp in whole cell lysates, suggesting that ω3PUFAs do not modulate the expression of Pgp, but only its intracellular distribution. To our knowledge, the shift of MRP1 from DRMs into cytosol following cholesterol depletion has not been described yet. In HT29-dx cells, the surface level of MRP1 was also decreased by ω3PUFAs, suggesting that the localization of this transporter may be sensitive as well to the amount of cholesterol present in the plasma-membrane.

Beside lowering the amount of cholesterol, PUFAs were effectively incorporated in whole cells and DRMs of HT29 and HT29-dx cells even if with different efficiency. Recent works performed on reconstructed membranes and B lymphocytes described a higher incorporation of DHA compared to EPA in whole cell lipids, in detergent-soluble membranes and DRMs [55-57]. In HT29 cells, whereas EPA and DHA were inserted at a similar extent in DRMs, EPA was more greatly incorporated than DHA in whole cell lipids. Such discrepancy with the situation observed in B lymphocytes may be due to different protocols of treatment and different metabolism of ω3PUFAs in the two cell types [46]. For instance, after 15 hours of treatment, an higher increase of DPA, a metabolite derived from EPA, was observed in B lymphocytes [56]. Interestingly, the sum of EPA and DPA incorporated in B lymphocyte membranes [56] is in the same range of EPA incorporation measured in HT29 and HT29-dx cells, wherein EPA is poorly metabolized into DPA. This is not surprising, because different cell types have different rate of uptake, metabolism and incorporation of ω3PUFAs [46]. It is conceivable that the enzymatic activity of the elongase which metabolizes EPA into DPA is low in our cell lines, making the availability of EPA and its consequent incorporation in cell membranes higher than in other cell models.

There is a general agreement that DHA actively modifies the DRMs organization, producing DRMs disassembly and cholesterol displacement, inducing lateral phase separation into PUFA-rich/sterol-poor microdomains and impairing the activity of many DRM-associated proteins [34,36]. Pgp is one of these proteins, since its activity is significantly reduced when the lipid environment of DRMs is altered [58]. Together with the effects on cholesterol synthesis, the incorporation of EPA and DHA in DRM phospholipids is a second mechanisms by which ω3PUFAs decrease the levels of Pgp and MRP1 in HT29-dx cells.

According to its structure, BCRP is less hydrophobic than Pgp and MRP1 [27]. We cannot exclude that – differently from what we observed for Pgp and MRP1 – the enrichment of ω3PUFAs and ω6PUFAs may favor the retention of BCRP in DRMs compartment. In particular the enrichment of DHA seemed the most suitable condition to increase BCRP levels in DRMs, in both HT29 and HT29-dx cells. Since the first-line drugs used in the treatment of colon cancer (e.g. irinotecan, oxaliplatin, 5-fluorouracil) are not substrates of BCRP, we do not believe that the increase of BCRP may negatively affect the outcome of patients subjected to the standard chemotherapeutic regimen for colon cancer.

By contrast, in consequence of the decreased surface levels of Pgp and MRP1, DHA and EPA overcame chemoresistance to doxorubicin, a substrate of these two transporters [27], and to irinotecan, a substrate of Pgp [44]. A similar effect was produced by DHA and EPA in the Pgp-overexpressing Caco-2 cells treated with paclitaxel [59]. DHA and EPA fully restored both the direct cytotoxicity and the “indirect” pro-immunogenic toxicity of doxorubicin, i.e. they decreased the cell viability, and increased the release of ATP and HMGB1, the translocation of calreticulin and the phagocytosis. Calreticulin expression is usually associated with good prognosis in patients with advanced-stage colon cancer, and correlates with a proper antitumor response of the host immune system [60]. We demonstrated that the association of ω3PUFAs and doxorubicin enhanced the exposure of calreticulin in colon chemoresistant cells. Although doxorubicin is not currently used in colon cancer therapy, our work suggests that the combination of ω3PUFAs and doxorubicin might be noteworthy of further studies to set up effective chemo-immunotherapy protocols in colon cancer, a disease in which immune-based interventions are under clinical investigation [61,62]. Irinotecan is currently used in colon cancer treatment. Our results, showing that DHA and EPA lowered the IC50 of irinotecan and completely restored its anti-proliferative effects in HT29-dx cells, may have a translational potential in clinical settings.

The chemosensitizing effect of DHA and EPA has been already described in HT29 cells, where they enhance the pro-apoptotic effects of irinotecan, oxaliplatin and 5-fluorouracil [63]. Our study is the first describing the efficacy of ω3PUFAs in chemoresistant colon cancer cells. The mechanism that we propose for such chemosensitization – i.e. the inhibition of ABC transporters activity – might help to explain why a dietary supplementation with ω3PUFAs improves the efficacy of irinotecan in mice bearing colon cancer [64], the efficacy of anthracyclines in patients with breast cancer [65], the efficacy of cisplatin plus vinorelbine in patients with non small cell lung cancer [66]. Moreover our work provides the biochemical mechanism explaining why vincristine, a substrate of Pgp and MRP1, was less effluxed by drug-resistant human cervical cancer KB-Ch^R^-8-5 cells treated with ω3PUFAs [67].

## Conclusions

We describe here a new effect of ω3PUFAs, i.e. the down-regulation of the endogenous cholesterol synthesis by promoting HMGCoAR ubiquitination via Trc8 E3 ligase. Such effect is particularly pronounced in cells with a dysregulated cholesterol synthesis – i.e. with high demand of cholesterol, high intracellular levels of cholesterol, high amount of HMGCoAR – such as MDR cells. The decreased amount of cholesterol and the increased incorporation of ω3PUFAs in DRMs alter the physicochemical properties of these compartments that are essential for the proper localization and activity of ABC transporters. As a result of these events, ω3PUFAs overcome drug resistance towards substrates of Pgp and MRP1, and restore a proper tumor-immune system recognition in response to chemotherapy in MDR cells (Additional file 5).

The effects of ω3PUFAs are likely not limited to colon cancer cells, because the increased demand of cholesterol is a typical feature of several types of chemoresistant tumors [25,26].

A diet rich of saturated fatty acids increases, whereas a diet rich of ω3PUFAs decreases the onset of colon cancer. Whereas the chemopreventing effects of ω3PUFAs are well-documented in colon cancer [11-16], their effects as MDR-overcoming agents have not been reported previously in this tumor. Compared with other chemosensitizing agents affecting the synthesis of cholesterol – e.g. statins, aminobisphosphonates – [25,26], ω3PUFAs are safer and have less side-effects. Therefore, a dietetic supplementation with ω3PUFAs may be regarded not only as a useful chemopreventive strategy, but also as a potential adjuvant approach in patients with colon cancers unresponsive to chemotherapy.

## Methods

### Chemicals

Fetal bovine serum (FBS) and culture medium were purchased from Invitrogen Life Technologies (Carlsbad, CA). Plasticware for cell cultures was from Falcon (Becton Dickinson, Franklin Lakes, NJ). AA, DHA, EPA, doxorubicin, irinotecan were from Sigma Chemical Co. (St. Louis, MO). Stock solutions of PUFAs were dissolved in absolute ethanol and added on cell cultures as a mixture PUFAs/ fatty acid free bovine serum albumin (BSA), with a 3:1 ratio. The amount of ethanol added in each dish never exceeded 0.5% v/v. Control cells were treated with a 0.5% v/v solution of ethanol. Simvastatin and MG-132 were obtained from Calbiochem (San Diego, CA). Electrophoresis reagents were from Bio-Rad Laboratories (Hercules, CA). The protein content of cell monolayers and lysates was assessed with the BCA kit from Sigma Chemical Co. Unless otherwise specified, all the other reagents were from Sigma.

### Cells

Human chemosensitive colon cancer HT29 cells were from ATCC (Rockville, MD). The chemoresistant counterpart (HT29-dx cells) line was generated by culturing parental cells in the presence of increasing concentrations of doxorubicin for 20 passages [41]. HT29-dx cells have higher Pgp, MRP1 and BCRP than HT29 cells [68]; moreover, compared to HT29 cells, HT29-dx cells have a higher IC50 for doxorubicin (25.51 ± 1.33 μM versus 2.74 ± 0.67 μM; p < 0.01), irinotecan (0.91 ± 0.18 μM versus 0.05 ± 0.01 μM; p < 0.001), oxaliplatin (98.75 ± 6.08 μM versus 4.75 ± 0.71 μM; p < 0.001), and 5-fluorouracil (8.35 ± 0.64 μM versus 1.06 ± 0.14 μM; p < 0.05), representing a reliable model of MDR cells. For the present work, HT29-dx cells were grown in medium containing 200 nM doxorubicin. Cell cultures were maintained in RPMI-1640 medium supplemented with 10% v/v FBS, 1% v/v penicillin-streptomycin and 1% v/v L-glutamine, in a humidified atmosphere at 37°C and 5% CO_2_. All the experiments with these cell lines were approved by Bioethics Committee (“Comitato di Bioetica d’Ateneo”) of the University of Torino, Italy.

### De novo synthesis of cholesterol

Cells were labeled with 1 μCi/ml [^3^H]acetate (3600 mCi/mmol; Amersham Bioscience, Piscataway, NJ) and the synthesis of radiolabeled cholesterol was measured as described [69]: after 24 h the cells were washed twice with phosphate-buffered saline (PBS) and mechanically scraped in 200 μl of PBS. 500 μl of methanol and 1 ml of hexane were added to the cell suspension, which was stirred at room temperature for 1 h and then centrifuged at 2,000 × g for 5 min. The upper phase containing hexane was transferred into a new test tube, and the lower phase was supplemented with 1 ml of hexane and stirred overnight. After a 5 min centrifugation at 2,000 × g, the upper phase was added to the previous one and the solvent was allowed to evaporate at room temperature for 24 h. Cellular lipid extracts produced by this separation were re-suspended in 30 μl of chloroform and then subjected to thin layer chromatography (TLC), using a 1:1 (v/v) ether/hexane solution as mobile phase. Each sample was spotted on pre-coated LK6D Whatman silica gels (Merck, Darmstadt, Germany) and allowed to run for 30 min. Solutions of 10 μg/ml cholesterol were used as standard. The silica gel plates were exposed for 1 h to a iodine-saturated atmosphere, then the migrated spots were cut out and their radioactivity was measured by liquid scintillation, using a Tri-Carb Liquid Scintillation Analyzer (PerkinElmer, Waltham, MA). The results were expressed as fmol [^3^H]cholesterol/mg cell proteins, according to the relative calibration curve.

### Cells viability, cytotoxicity and proliferation assays

In viability assays, cells were seeded in 24-wells plates, treated for 24 h with AA, DHA and EPA at 25, 50, 100, 200 μM, then incubated for 1 h at 37°C in culture medium containing 70 μg/ml of Neutral Red solution (Sigma). Cells were washed three times with PBS and rinsed with stop buffer (32 mM trisodium citrate, 50% v/v methanol; pH 6). The absorbance at 540 nm was read using a Synergy HT Multi-Detection Microplate Reader (Bio-Tek, Winooski, VT). The absorbance of untreated cells was considered as 100% viability; the results were expressed as percentage of viable cells versus untreated cells.

The percentage of annexin-V-positive cells, considered as apoptotic cells, was measured as previously reported [69]: cells were treated for 24 h with 50 μM AA, DHA or EPA, washed twice with fresh PBS, detached with 200 μl of Cell Dissociation Solution (Sigma) for 10 min at 37°C and re-suspended in 500 μl of binding buffer (100 mM Hepes, 140 mM NaCl, 25 mM CaCl_2_, pH 7.5). Each sample was incubated with 10 μM Annexin V-fluorescein isothiocyanate (FITC) for 5 min at room temperature and the fluorescence was recorded using a FACSCalibur system (Becton Dickinson Biosciences, San Jose, CA), with a 530 nm band pass filter. For each analysis 10,000 events were collected and the percentage of cells positive for Annexin V-FITC was calculated by the Cell Quest software (Becton Dickinson Biosciences).

The extracellular release of high-mobility group 1 box (HMGB1) protein was taken as index of necrotic and immunogenic death, the extracellular release of ATP was considered an index of immunogenic death. To measure the extracellular release of HMGB1, 20 μl of the cell culture medium were boiled, resolved by SDS-PAGE and probed with an anti-HMGB1 antibody (Sigma). Blots were pre-stained with Red Ponceau to check the equal loading of proteins. The ATP release was measured on 100 μl of the cell culture medium with the ATP Bioluminescent Assay Kit (FL-AA, Sigma Aldrich Co.), using a Synergy HT Multi-Detection Microplate Reader. The results were expressed as nmol ATP/mg protein, according to the titration curve previously set.

In proliferation assays, 10,000 cells were seeded in 96-wells plates and treated with 1 μM irinotecan, 50 μM DHA, 50 μM EPA, alone or in co-incubation. At 12, 24, 48, 72, 96 h, cells were fixed with 4% w/v paraformaldehyde and stained with 0.5% w/v crystal violet solution for 10 min at room temperature. The plate was washed three times in water, then 100 μl of 0.1 mM sodium citrate in 50% v/v ethanol was added to each well. The absorbance was read at 570 nm using a Synergy HT Multi-Detection Microplate Reader. The absorbance units were converted into number of cells, according to a titration curve obtained with serial cells dilutions.

### HMGCoAR activity

The activity of HMGCoAR was measured in microsomal fractions as described previously [69]. The cells were rinsed with the lysis buffer (10 mM Tris, 100 mM NaCl, 20 mM KH_2_PO_4_, 30 mM EDTA, 1 mM EGTA, 250 mM sucrose, pH 7.5) supplemented with protease inhibitor cocktail set III (100 mM AEBSF, 80 mM aprotinin, 5 mM bestatin, 1.5 mM E- 64, 2 mM leupeptin,1 mM pepstatin; Calbiochem), 1 mM Na_3_VO_4_, 1 mM NaF, 1 mM 4-(2-aminoethyl)benzenesulphonyl fluoride (PMSF), 10 mM aprotinin, 10 mM dithiothreitol (DTT). After sonication (2 bursts of 10 s; Labsonic sonicator, Sartorius Stedim Biotech S.A., Aubagne Cedex, France), cell lysates were centrifuged at 13,000 × g for 15 min at 4°C; the supernatants were subjected to ultracentrifugation at 100,000 × g for 1 h at 4°C, using a Optima L-90 K Beckman Coulter Ultracentrifuge (Beckman Coulter Inc, Fullerton, CA) to collect the microsomal fraction, which was re-suspended in 250 μl of lysis buffer and stored at - 80°C until the use. 12.5 μg of microsomal protein extracts, re-suspended in 25 μl, were supplemented with 10 mM DTT, 5 mM NADP and with a NADPH-generating system (1.3 mM glucose 6-phosphate, 0.67 U/ml of glucose-6-phosphate dehydrogenase, 33 mM MgCl_2_). The reaction was started by adding 60 nCi [^14^C]HMG-CoA (50-62 mCi/mmol, Amersham Bioscience). After a 20 min incubation at 37°C the reaction was stopped with 25 μl of 10 N HCl. The samples were stirred for 30 min at 37°C to enhance the complete lactonization of mevalonic acid, centrifuged at 13,000 × g for 2 min and separated by TLC on silica gel plates with hexane/acetone (1:1, v/v) as mobile phase. A 1 mM solution of purified mevalonolactone was used as standard. The labeled product (^14^C-mevalonolactone) was recovered from the TLC plates and quantified by liquid scintillation. The results were expressed as nmol [^14^C]HMGCoA/min/mg cell proteins, according to the relative calibration curve.

### Quantitative Real Time-PCR (qRT-PCR)

Total RNA was extracted and reverse-transcribed using the QuantiTect Reverse Transcription Kit (Qiagen, Hilden, Germany). RT-PCR was carried out with IQ™ SYBR Green Supermix (Bio-Rad). The same cDNA preparation was used for the quantitation of *HMGCoAR*, *HMGCoAS* and *actin*, chosen as a housekeeping gene. The sequences of primers were: *HMGCoAR*: 5′-CGCAACCTCTATATCCGT-3′; 5′-GTAGCCGCCTATGCTC-3′; *HMGCoAS*: 5′-TTGGTAGTTGCAGGAGACATCGCT-3′; 5′-AGCATTTGGCCCAATTAGCAGAGC-3′; *actin*: 5′-GCTATCCAGGCTGTGCTATC-3′; 5′-TGTCACGCACGATTTCC-3′. The relative quantitation of each sample was performed by comparing each PCR product with the housekeeping PCR product, using the Software Gene Expression Quantitation (Bio-Rad).

### Western blot analysis

For total, phosphorylated or ubiquitinated HMGCoAR Western blot analysis, 50 μg of microsomal proteins, collected as reported above, were immunoprecipitated with an anti-HMGCoAR antibody (Santa Cruz Biotechnology, Santa Cruz, CA), in the presence of 100 mM DTT and 1 mM mevalonic acid. For total HMGCoAR, the immunoprecipitated samples were separated by SDS-PAGE, transferred to polyvinylidene fluoride membrane sheets (Immobilon-P, Millipore, Bedford, MA) and probed with an anti-HMGCoAR antibody, followed by the peroxidase-conjugated secondary antibody (Bio-Rad). To detect HMGCoAR phosphorylated on serine, blots were incubated with a biotinylated anti-phosphoserine antibody (Sigma), followed by a streptavidin/horseradish peroxidase-conjugated polymer (Sigma). For ubiquitinated HMGCoAR, blots were probed with an anti-ubiquitin antibody (Enzo Life Science, Farmingdale, NY), followed by the peroxidase-conjugated secondary antibody. The levels of calreticulin, detected by a specific antibody (ABR Affinity Bioreagents, Thermo Scientific, Waltham, MA), were measured to check to equal loading of microsomal proteins. Blot proteins were detected by enhanced chemiluminescence (PerkinElmer).

To analyze the expression of Insig-1, Insig-2, gp78/AMFR, Trc8/RNF-139, 20 μg of the microsomal extracts were probed with the following antibodies: anti-Insig-1 (Abcam, Cambridge, MA), anti-Insig-2 (Abcam), anti-gp78/AMFR (GeneTex Inc., Irvine, CA), anti-Trc8/RNF-139 (Abnova, Taipei City, Taiwan).

Whole cell lysates were used for the Western blot analysis of Pgp, MRP1, BCRP. Cells were lysed in Mg^2+^ Lysis/Wash Buffer (125 mM Tris-HCl, 750 mM NaCl, 1% v/v NP40, 10% v/v glycerol, 50 mM MgCl_2_, 5 mM EDTA, 25 mM NaF, 1 mM Na_3_VO_4_, 10 μg/ml leupeptin, 10 μg/ml pepstatin, 10 μg/ml aprotinin, 1 mM PMSF, pH 7.5), sonicated and centrifuged at 13,000 × g for 10 min at 4°C. 30 μg cell lysates were subjected to Western blotting with anti-Pgp (Calbiochem), anti-MRP1 (Abcam), anti-BCRP (Santa Cruz Biotechnology Inc.), anti-tubulin (Santa Cruz Biotechnology Inc.) antibodies. For DRMs samples, 5 μg of proteins from DRM fractions (see below) were probed in Western blot analysis with anti-Pgp, anti-MRP1, anti-BCRP, anti-flotillin (Abcam) or anti-clathrin heavy chain (Abcam) antibodies.

Nucleus-cytosol separation was performed by using the Nuclear Extract kit (Active Motiv, La Hulpe, Belgium). 10 μg of nuclear extracts were subjected to Western blot analysis, using anti-SREBP-1, anti-SREBP-2 or anti-proliferating cell nuclear antigen (PCNA) antibody (Santa Cruz Biotechnology Inc.).

The densitometric analysis of Western blots was performed with the ImageJ software ( http://rsb.info.nih.gov/ij/). Results were expressed as arbitrary units, where ‘1 unit’ is the mean band density in untreated cells.

### HMGCoAR ubiquitination assay

To measure the ubiquitination of HMGCoAR, microsomal compartments were isolated from cell as described above. The ubiquitination assay was performed using the E3Lite Customizable Ubiquitin Ligase kit (LifeSensors Inc., Malvern, PA): 100 μg of microsomal proteins were diluted in 100 μl of ubiquitination assay buffer (1 M Tris/HCl, 500 mM MgCl_2_, 10 mM DTT, pH 8), and incubated for 30 min at 37°C, in the presence of 5 nM E1 activating enzyme provided by the kit, 100 nM E2 conjugating enzyme Ube2g2 (LifeSensors Inc.), 200 μM ATP, 6 mM human recombinant ubiquitin. When indicated, 1 μM AA, DHA or EPA were added. Samples were washed twice with PBS-Tween 0.1% v/v containing 5% w/v BSA and immunoprecipitated with the anti-HMGCoAR antibody. The immunoprecipitated samples were then incubated with the biotinylated anti-ubiquitin antibody of the kit, followed by the streptavidin/horseradish peroxidase-conjugated polymer and enhanced chemiluminescence detection reagent. The chemiluminescent signal was read using a Synergy HT Multi-Detection Microplate Reader. A blank was performed in the absence of microsomal extracts and its luminescence was subtracted from the luminescence of each sample. The results were expressed as relative luminescence units (RLU)/mg of microsomal proteins.

To measure the ubiquitination of HMGCoAR in cell-free systems, 1 μg of human recombinant HMGCoAR (catalytic domain, GST-fusion protein produced in *E. coli*, Sigma), dissolved in 100 μl of ubiquitination assay buffer, was incubated with 5 nM E1 activating enzyme provided by the E3Lite Customizable Ubiquitin Ligase kit, 100 nM E2 conjugating enzyme Ube2g2, 200 μM ATP, 6 mM human recombinant ubiquitin, 1.5 μM human recombinant E3 ligases Trc8/RNF-139 (Abnova) or gp78/AMFR (Abnova). AA, DHA or EPA were added at different concentrations. Samples were maintained at 37°C for 30 min; the quantification of ubiquitinated HMGCoAR was performed as reported above. This assay was considered an index of the activity of Trc8 and gp78 E3 ligases, respectively.

### Lipid composition analysis

Lipids were extracted according to [70] with three different chloroform/methanol mixtures (1:1, 1:2 and 2:1, v/v) and partitioned with water and with the theoretical upper phase (chloroform/methanol/water, 47:48:1, v/v). The organic phase was dried and then suspended in chloroform/methanol (2:1, v/v) for the analysis of total fatty acid and cholesterol contents. Each solvent contained 50 μM 2,6-bis(1,1-dimethylethyl)-4-methylphenol (BHT).

Total fatty acids were determined as methyl esters by gas chromatography. The methyl esters were obtained by reaction with 3.33% (w/v) sodium methoxide in methanol and injected into an Agilent Technologies (6850 series II) gas chromatograph equipped with a flame ionization detector and a capillary column (AT Silar) (length 30 m, film thickness 0.25 μm). The carrier gas was helium, the injector temperature was 250°C, the detector temperature was 275°C, the oven temperature was set at 50°C for 20 min and then increased to 200°C at 10°C min^–1^ for 20 min. A standard mixture containing all fatty acid methyl esters (Sigma) was injected for calibration, and heptadecanoic acid methyl ester (Sigma) was used as internal standard.

The cholesterol analysis was achieved by high-pressure liquid chromatography HPLC (Jasco, Tokyo, Japan) equipped with ELSD detector (Sedere, Alfortville Cedex, France) and silica normal-phase LiChrospher Si 60 column (LiChroCART 250-4; Merck, Darmstadt, Germany). The cholesterol amount was related to the cell protein content.

### Isolation of Detergent Resistant Membranes (DRMs)

The extraction of DRMs was performed as reported previously [51]. This is a common biochemical method to analyze the domain organization of membranes. Although detergent treatment disrupts most lipid-lipid interactions, a minor fraction of cell membranes is preserved and can be isolated as DRMs. DRMs represent no-native “lipid rafts” but they are a useful tool to study changes in composition related to biochemical, physiological or pathological raft associated events [71].

Cells were harvested by scraping in PBS containing 0.4 mM Na_3_VO_4_, then centrifuged at 1,300 × g for 2 min, suspended in 1.4 ml lysis buffer (1% Triton X-100, 10 mM Tris buffer, pH 7.5, 150 mM NaCl, 5 mM EDTA, 1 mM Na_3_VO_4_, 1 mM PMSF, 75 mU/ml aprotinin), maintained in ice for 20 min, and finally treated with Dounce homogenizer (10 strokes, tight). The DRMs were purified on sucrose gradient as previously described [35]. After centrifugation 11 fractions were collected; to confirm the purity of DRMs, the content of cholesterol, phospholipids, gangliosides, clathrin heavy chain and flotillin-1 was determined in each fraction by TLC and Western blot (data not shown). DRMs protein content, fatty acid composition and cholesterol content were analyzed as described above.

### Flow cytometry analysis

Cells were washed twice with PBS, rinsed with 1 ml of 0.25% (w/v) PBS-BSA and centrifuged at 10,000 × g for 5 min. The pelleted cells were incubated for 45 min at 4°C with the anti-Pgp, anti-MRP1, anti-BCRP or anti-calreticulin antibodies, then washed and incubated with a secondary FITC-conjugated antibody for 30 min at 4°C in the dark. After fixation in paraformaldehyde 2% v/v, cells were re-suspended in 0.5 ml PBS-BSA and analyzed using a FACS-Calibur system. For each analysis 100,000 events were collected. The percentage of viable (propidium iodide-negative) fluorescent cells was calculated by the Cell Quest software. Control experiments included incubation with non immune isotypic antibodies followed by the secondary antibody.

### Intracellular doxorubicin accumulation

Intracellular doxorubicin content was detected with a fluorimetric assay as reported [41]. Cells were incubated for 24 h in medium containing 5 μM doxorubicin, washed twice with PBS, detached with trypsin/EDTA, centrifuged for 30 s at 13,000 × g, re-suspended in 1 ml of a 1:1 mixture of ethanol/0.3 N HCl and sonicated. The protein content of the cell lysates was measured and the amount of intracellular doxorubicin was detected using a PerkinElmer LS-5 spectrofluorimeter (PerkinElmer). Excitation and emission wavelengths were 475 and 553 nm, respectively. A blank was prepared in the absence of cells in each set of experiments and its fluorescence was subtracted from the one measured in each sample. The results were expressed as nmol doxorubicin/mg cell proteins according to a previously prepared calibration curve.

### Dendritic cell (DC) generation and in vitro phagocytosis assay

The generation of DCs and their phagocytosis of colon cancer cells were performed as described earlier [26]. DCs were generated from peripheral blood samples obtained from healthy donors kindly provided by the local Blood Bank (Fondazione Strumia, Torino, Italy). Cells were harvested on day 6 and confirmed as immature DCs by morphology and immunophenotype (data not shown). HT29 and HT29-dx cells were green-stained with PKH2-FITC (Sigma), washed twice and incubated with DCs at a ratio of 1:1 for 18 h at 37°C. Co-cultures were then stained for 20 min at 4°C with APC-conjugated HLA-DR antibody (Miltenyi Biotec, Tetrow, Germany) to mark DCs. Two-color flow cytometry was performed with FACS-Calibur system and CellQuest software. At least 10,000 events were accumulated specifically backgating on DC morphology (region 1: FSC versus SSC). Tumor cell phagocytosis was assessed as the percentage of double-stained (FITC plus APC) cells. Tumor cells do not express significant amounts of HLA-DR, and they were excluded from region 1 by their morphology. In each set of experiments, a phagocytosis assay was performed by co-incubating DCs and tumor cells at 4°C, instead of 37°C, and the percentage of double-stained cells obtained after the incubation at 4°C was subtracted from values observed at 37°C. The phagocytosis rate was expressed as “phagocytic index”, calculated as reported in [42].

### Statistical analysis

Data are reported as mean ± SD of at least three independent experiments. Results were checked for normal distribution and analyzed by a one-way analysis of variance (ANOVA) followed by Dunnet’s t test. p < 0.05 was considered significant.

## Abbreviations

ω3/ω6PUFAs: Omega 3/omega 6 polyunsaturated fatty acids; DHA: Docosahexaenoic acid; EPA: Eicosapentaenoic acid; HDL: High density lipoprotein; LDL: Low density lipoprotein; HMGCoAR: 3-hydroxy-3-methylglutaryl-coenzyme a reductase; SREBP: Sterol regulatory element binding protein; HMGCoAS: 3-hydroxy-3-methylglutaryl-coenzyme a synthase; MDR: Multidrug resistance; ABC: ATP binding cassette; Pgp: P-glycoprotein; MRP: Multidrug resistance related protein; BCRP: Breast cancer resistance protein; DRMs: Detergent resistant membranes; AA: Arachidonic acid; DPA: Docosapentaenoic acid; FBS: Fetal bovine serum; BSA: Bovine serum albumin; PBS: Phosphate-buffered saline; TLC: Thin layer chromatography; FITC: Fluorescein isothiocyanate; HMGB1: High-mobility group 1 box; PMSF: 4-(2-aminoethyl)benzenesulfonyl fluoride; DTT: Dithiothreitol; PCNA: Proliferating cell nuclear antigen; RLU: Relative luminescence unit; HPLC: High-pressure liquid chromatography; DC: Dendritic cell; ER: Endoplasmic reticulum; ERAD: ER-associated degradation; HIF-1α: Hypoxia inducible factor-1α; SFA: Saturated fatty acids; MUFA: Mono-unsaturated fatty acids.

## Competing interests

The authors declare that they have no competing interests.

## Authors’ contributions

GG and PAC performed the cell viability assays, immunoassays and lipid analysis experiments; IC carried out the metabolic radiolabelling assays; GM participated in the lipid analysis experiments; JK, BC and EG carried out the flow cytometric analysis, the phagocytosis assays and the experiments with chemotherapeutic drugs; DG participated in the design of the study and analyzed the data; AMR and CR conceived of the study, analyzed the data and wrote the manuscript. All authors read and approved the final manuscript.

## Authors’ information

JK is the recipient of a “Mario e Valeria Rindi” fellowship from Italian Foundation for Cancer Research (FIRC).

## Supplementary Material

Additional file 1 **Effects of ω3PUFAs on apoptosis and necrotic/immunogenic death of colon cancer cells.** HT29 and HT29-dx cells were incubated for 24 h in the absence (CTRL) or presence of 50 μM arachidonic acid (AA), docosahexaenoic acid (DHA), eicosapentaenoic acid (EPA). Cycloheximide (4 μM for 24 h, CHX) was chosen as positive control of cytotoxicity in both chemosensitive and chemoresistant cells; doxorubicin (5 μM for 24 h, DOX) was chosen as positive control of cytotoxicity in chemosensitive cells. A. Annexin V staining. The percentage of cells positive for surface annexin V, taken as index of apoptosis, was measured in duplicate by flow cytometry. Data are presented as means ± SD (n = 3). Versus respective CTRL: * p < 0.001; DOX in HT29-dx versus DOX in HT29: ° p < 0.001. B. Western blot analysis of extracellular HMGB1, taken as index of necrotic/immunogenic death. Red Ponceau staining was used to check the equal loading of proteins. The figure is representative of two experiments with similar results. The band density ratio between HMGB1 and the Red Ponceau-positive bands was expressed as arbitrary units. Versus CTRL HT29: * p < 0.002. Click here for file

Additional file 2 **Effects of ω3PUFAs on *HMGCoAS *transcription and SREBPs nuclear translocation in colon cancer cells.** HT29 and HT29-dx cells were incubated for 24 h in the absence (CTRL) or presence of 50 μM arachidonic acid (AA), docosahexaenoic acid (DHA), eicosapentaenoic acid (EPA). A. Total RNA was extracted, reverse-transcribed and subjected to qRT-PCR for *HMGCoAS* gene. Measurements were performed in triplicate and data are presented as means ± SD (n = 3). Versus CTRL HT29: * p < 0.05. B. Western blot detection of SREBP2 and SREBP1, performed on nuclear extracts. Proliferating cell nuclear antigen (PCNA) expression was used as a control of equal loading of nuclear proteins. The figure is representative of three experiments with similar results. The band density ratio between each protein and PCNA was expressed as arbitrary units. Versus CTRL HT29: * p < 0.02. Click here for file

Additional file 3 **Effects of ω3PUFAs on Pgp, MRP1 and BCRP expression in colon cancer cells.** HT29 and HT29-dx cells were incubated for 48 h in the absence (CTRL) or presence of 50 μM arachidonic acid (AA), docosahexaenoic acid (DHA), eicosapentaenoic acid (EPA). The expression of Pgp, MRP1 and BCRP was measured on whole cell lysates by Western blotting. Tubulin expression was used as a control of equal protein loading. The figure is representative of three experiments with similar results. The band density ratio between each protein and tubulin was expressed as arbitrary units. Versus CTRL HT29: * p < 0.02. Click here for file

Additional file 4 **ω3PUFAs restore the pro-immunogenic death induced by doxorubicin in chemoresistant colon cancer cells.** HT29 and HT29-dx cells were incubated for 48 h in the absence (CTRL) or presence of 50 μM arachidonic acid (AA), docosahexaenoic acid (DHA), eicosapentaenoic acid (EPA). 5 μM doxorubicin (DOX) was added for 24 h, alone or during the last 24 h of incubation with fatty acids. Cycloheximide (4 μM for 24 h, CHX) was chosen as positive control of cytotoxicity in both chemosensitive and chemoresistant cells. A. The release of extracellular ATP was measured in triplicate by a chemiluminscent assay. Data are presented as means ± SD (n = 4). Versus respective CTRL: * p < 0.02; versus DOX alone: ° p < 0.01. D. Western blot analysis of extracellular HMGB1, taken as index of necrosis and immunogenic death. Red Ponceau staining was used to check the equal loading of protein. The figure is representative of two experiments with similar results. The band density ratio between HMGB1 and the Red Ponceau-positive bands was expressed as arbitrary units. Versus CTRL HT29: * p < 0.002; versus CTRL H29-dx: ° p < 0.002. Click here for file

Additional file 5 **Chemo-immunosensitizing effects of ω3PUFAs in chemoresistant colon cancer cells.** A. MDR cells such as HT29-dx have deficient activity of the Trc8 E3 ubiquitin ligase, higher expression and activity of 3-hydroxy-3-methylglutaryl-coenzyme A reductase, higher synthesis of cholesterol and higher levels of cholesterol in plasma-membrane. This situation favours the activity of ATP binding cassette transporters such as P-glycoprotein and limits the intracellular accumulation of specific chemotherapeutic drugs like doxorubicin, which is not able to induce direct cytotoxicity on tumor cell and to translocate calreticulin on cell surface, the first step to induce cell phagocytosis by dendritic cells. B. Docosahexaenoic acid and eicosapentaenoic acid restore the Trc8-mediated ubiquitnation of 3-hydroxy-3-methylglutaryl-coenzyme A reductase and its proteasomal degradation, lower the cholesterol synthesis and the amount of cholesterol in plasma-membrane and detergent resistant membranes. Moreover they are well incorporated in whole cell membrane and detergent resistant membranes, where they alter the physicochemical properties of the lipid environment and reduce the amount of P-glycoprotein. As a result, doxorubicin is more accumulated in MDR cells, exerts cytotoxic effects and promotes the surface translocation of calreticulin, followed by the dendritic cells-mediated phagocytosis. MDR: multidrug resistance; HMGCoAR: 3-hydroxy-3-methylglutaryl-coenzyme A reductase; Uq: ubiquitin; SREBP2: sterol regulatory element binding protein-2, Pgp: P-glycoprotein; CRT: calreticulin; d: doxorubicin; DHA: docosahexaenoic acid; EPA: eicosapentaenoic acid. Click here for file
