# Supplementary figures and images for: Omega 3 fatty acids chemosensitize multidrug resistant colon cancer cells by down-regulating cholesterol synthesis and altering detergent resistant membranes composition
Source: Mol Cancer. 2013 Nov 13;12:137. doi: 10.1186/1476-4598-12-137 (PMC4225767; doi:10.1186/1476-4598-12-137)

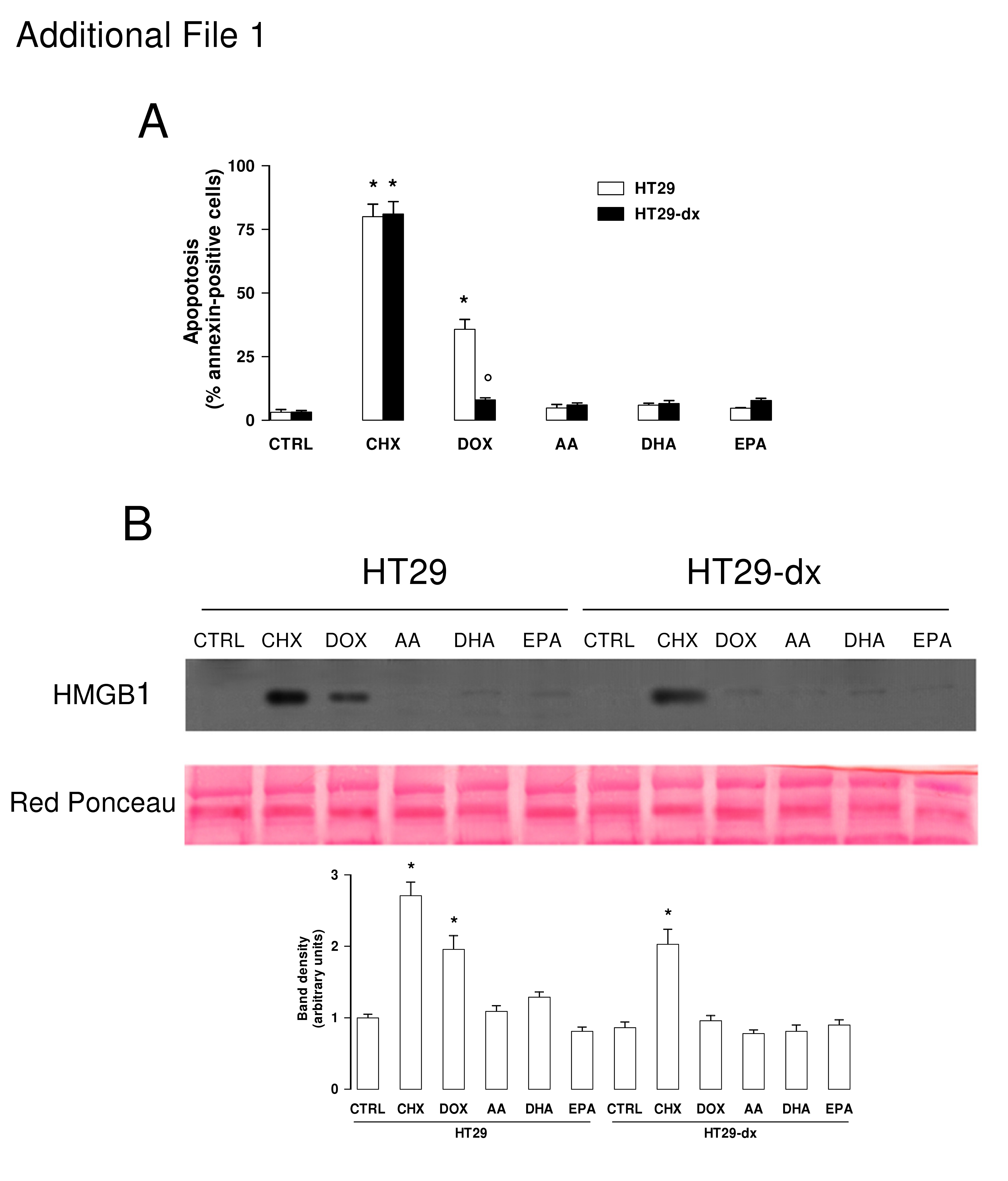

Supplement: Additional file 1 — Effects of ω3PUFAs on apoptosis and necrotic/immunogenic death of colon cancer cells. HT29 and HT29-dx cells were incubated for 24 h in the absence (CTRL) or presence of 50 μM arachidonic acid (AA), docosahexaenoic acid (DHA), eicosapentaenoic acid (EPA). Cycloheximide (4 μM for 24 h, CHX) was chosen as positive control of cytotoxicity in both chemosensitive and chemoresistant cells; doxorubicin (5 μM for 24 h, DOX) was chosen as positive control of cytotoxicity in chemosensitive cells. A. Annexin V staining. The percentage of cells positive for surface annexin V, taken as index of apoptosis, was measured in duplicate by flow cytometry. Data are presented as means ± SD (n = 3). Versus respective CTRL: * p < 0.001; DOX in HT29-dx versus DOX in HT29: ° p < 0.001. B. Western blot analysis of extracellular HMGB1, taken as index of necrotic/immunogenic death. Red Ponceau staining was used to check the equal loading of proteins. The figure is representative of two experiments with similar results. The band density ratio between HMGB1 and the Red Ponceau-positive bands was expressed as arbitrary units. Versus CTRL HT29: * p < 0.002. [file 1476-4598-12-137-S1.tiff]

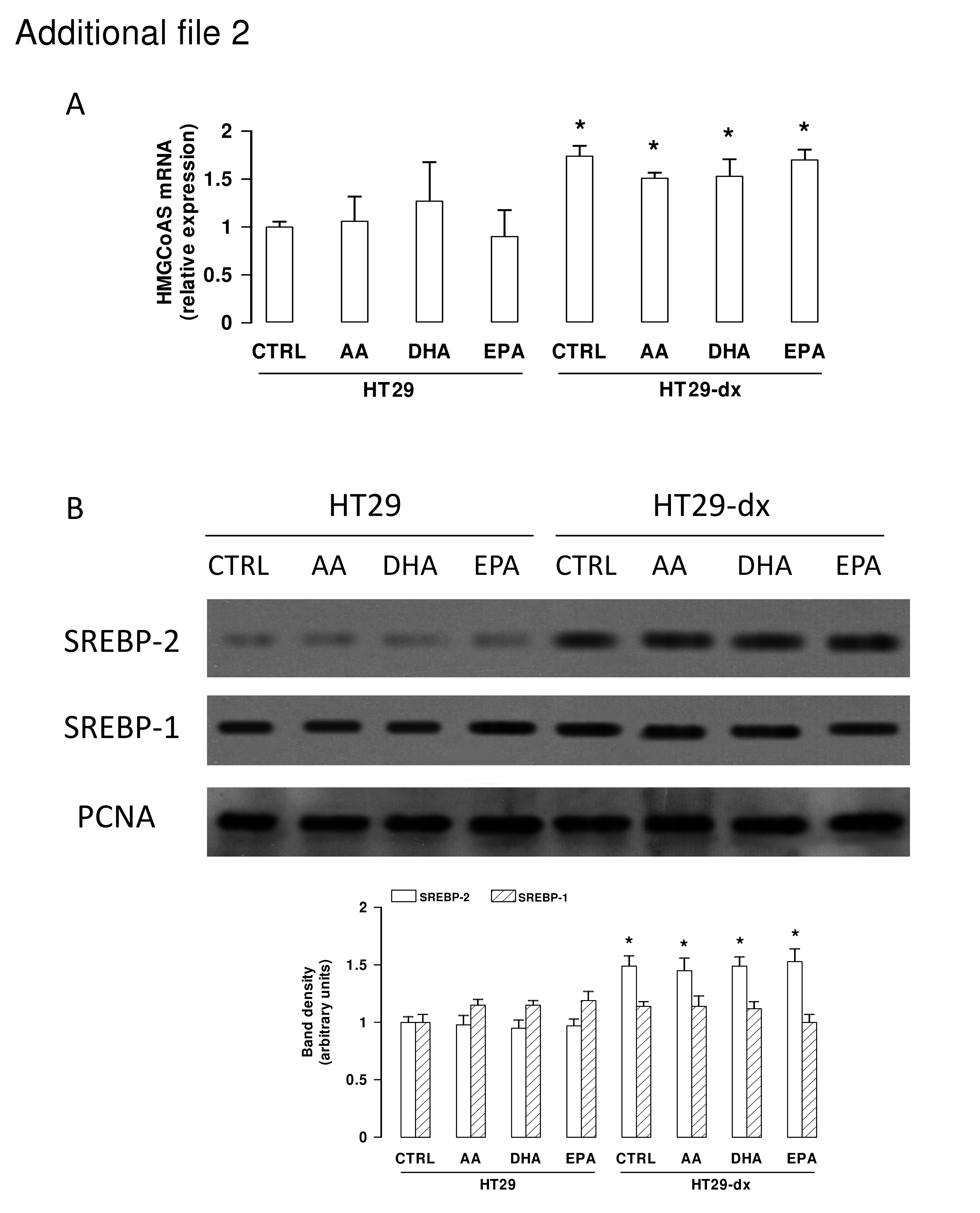

Supplement: Additional file 2 — Effects of ω3PUFAs on HMGCoAS transcription and SREBPs nuclear translocation in colon cancer cells. HT29 and HT29-dx cells were incubated for 24 h in the absence (CTRL) or presence of 50 μM arachidonic acid (AA), docosahexaenoic acid (DHA), eicosapentaenoic acid (EPA). A. Total RNA was extracted, reverse-transcribed and subjected to qRT-PCR for HMGCoAS gene. Measurements were performed in triplicate and data are presented as means ± SD (n = 3). Versus CTRL HT29: * p < 0.05. B. Western blot detection of SREBP2 and SREBP1, performed on nuclear extracts. Proliferating cell nuclear antigen (PCNA) expression was used as a control of equal loading of nuclear proteins. The figure is representative of three experiments with similar results. The band density ratio between each protein and PCNA was expressed as arbitrary units. Versus CTRL HT29: * p < 0.02. [file 1476-4598-12-137-S2.tiff]

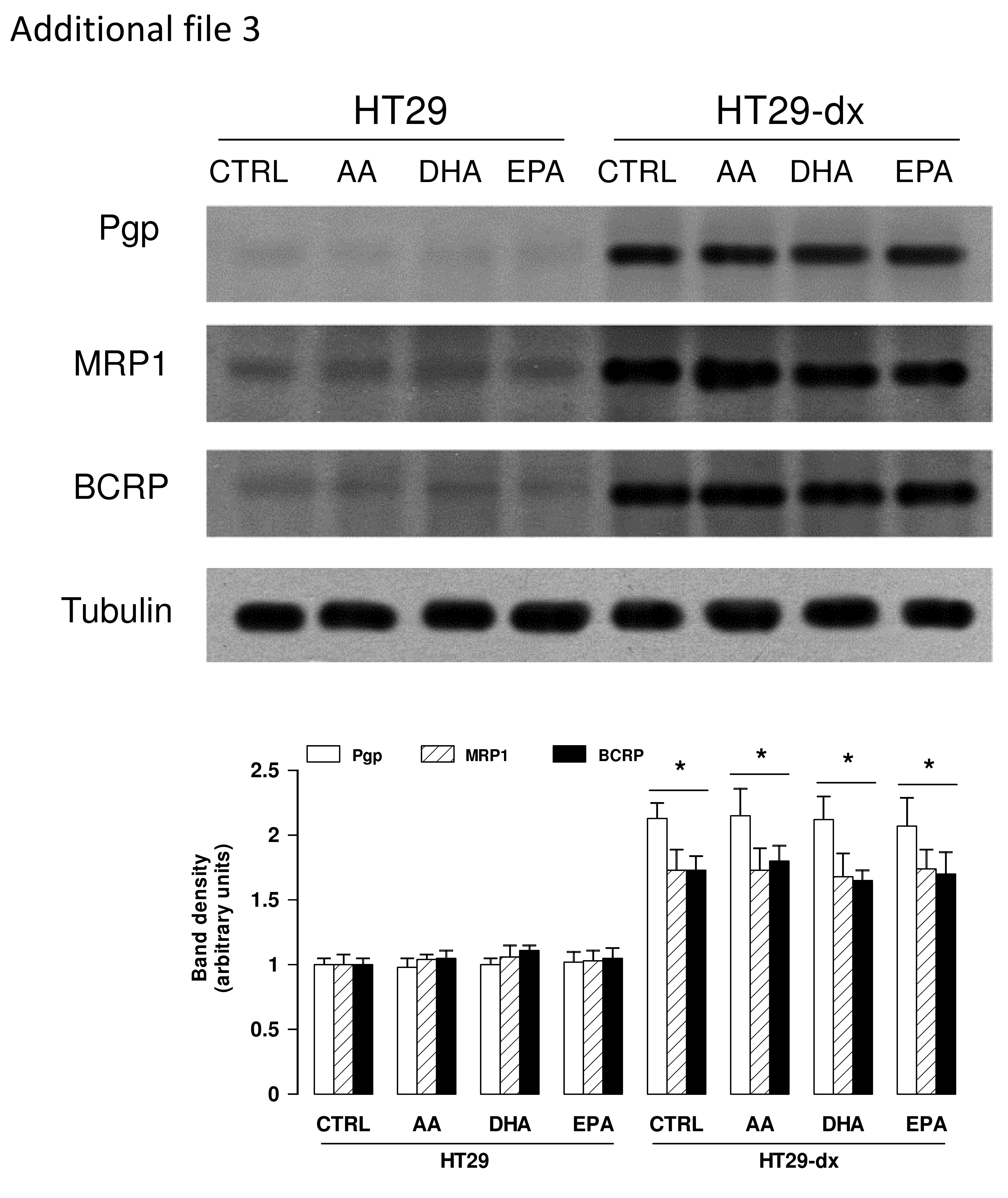

Supplement: Additional file 3 — Effects of ω3PUFAs on Pgp, MRP1 and BCRP expression in colon cancer cells. HT29 and HT29-dx cells were incubated for 48 h in the absence (CTRL) or presence of 50 μM arachidonic acid (AA), docosahexaenoic acid (DHA), eicosapentaenoic acid (EPA). The expression of Pgp, MRP1 and BCRP was measured on whole cell lysates by Western blotting. Tubulin expression was used as a control of equal protein loading. The figure is representative of three experiments with similar results. The band density ratio between each protein and tubulin was expressed as arbitrary units. Versus CTRL HT29: * p < 0.02. [file 1476-4598-12-137-S3.tiff]

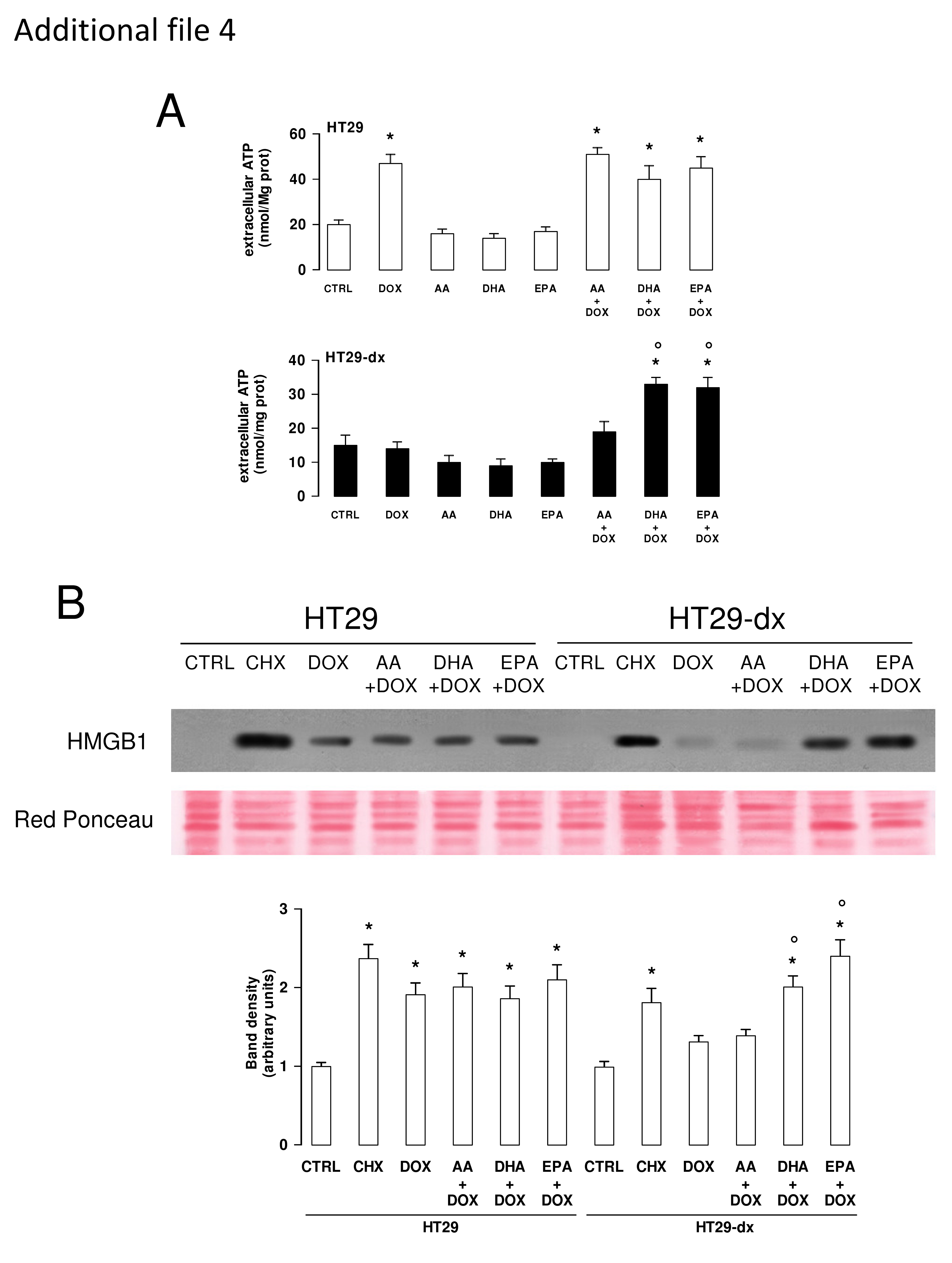

Supplement: Additional file 4 — ω3PUFAs restore the pro-immunogenic death induced by doxorubicin in chemoresistant colon cancer cells. HT29 and HT29-dx cells were incubated for 48 h in the absence (CTRL) or presence of 50 μM arachidonic acid (AA), docosahexaenoic acid (DHA), eicosapentaenoic acid (EPA). 5 μM doxorubicin (DOX) was added for 24 h, alone or during the last 24 h of incubation with fatty acids. Cycloheximide (4 μM for 24 h, CHX) was chosen as positive control of cytotoxicity in both chemosensitive and chemoresistant cells. A. The release of extracellular ATP was measured in triplicate by a chemiluminscent assay. Data are presented as means ± SD (n = 4). Versus respective CTRL: * p < 0.02; versus DOX alone: ° p < 0.01. D. Western blot analysis of extracellular HMGB1, taken as index of necrosis and immunogenic death. Red Ponceau staining was used to check the equal loading of protein. The figure is representative of two experiments with similar results. The band density ratio between HMGB1 and the Red Ponceau-positive bands was expressed as arbitrary units. Versus CTRL HT29: * p < 0.002; versus CTRL H29-dx: ° p < 0.002. [file 1476-4598-12-137-S4.tiff]

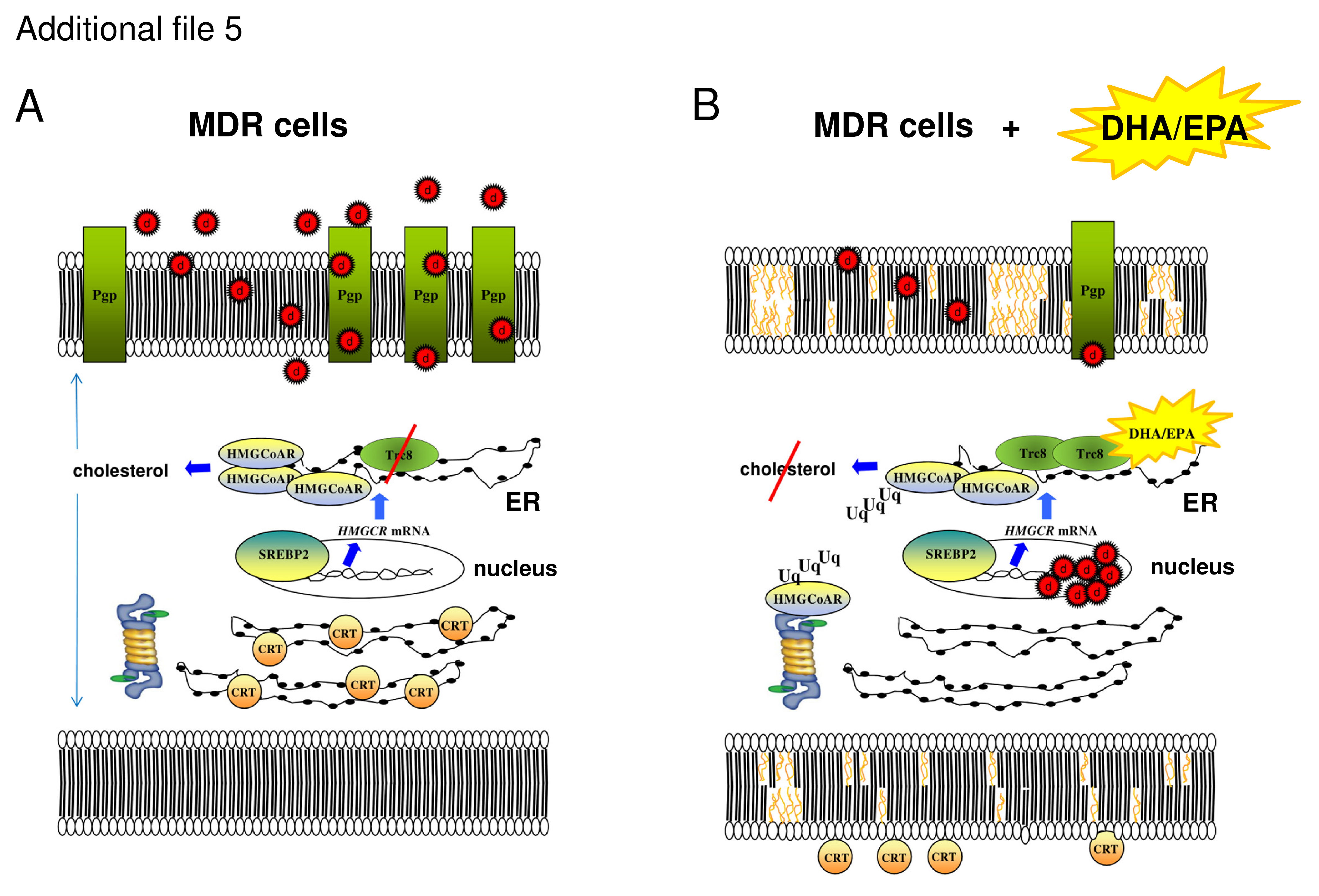

Supplement: Additional file 5 — Chemo-immunosensitizing effects of ω3PUFAs in chemoresistant colon cancer cells. A. MDR cells such as HT29-dx have deficient activity of the Trc8 E3 ubiquitin ligase, higher expression and activity of 3-hydroxy-3-methylglutaryl-coenzyme A reductase, higher synthesis of cholesterol and higher levels of cholesterol in plasma-membrane. This situation favours the activity of ATP binding cassette transporters such as P-glycoprotein and limits the intracellular accumulation of specific chemotherapeutic drugs like doxorubicin, which is not able to induce direct cytotoxicity on tumor cell and to translocate calreticulin on cell surface, the first step to induce cell phagocytosis by dendritic cells. B. Docosahexaenoic acid and eicosapentaenoic acid restore the Trc8-mediated ubiquitnation of 3-hydroxy-3-methylglutaryl-coenzyme A reductase and its proteasomal degradation, lower the cholesterol synthesis and the amount of cholesterol in plasma-membrane and detergent resistant membranes. Moreover they are well incorporated in whole cell membrane and detergent resistant membranes, where they alter the physicochemical properties of the lipid environment and reduce the amount of P-glycoprotein. As a result, doxorubicin is more accumulated in MDR cells, exerts cytotoxic effects and promotes the surface translocation of calreticulin, followed by the dendritic cells-mediated phagocytosis. MDR: multidrug resistance; HMGCoAR: 3-hydroxy-3-methylglutaryl-coenzyme A reductase; Uq: ubiquitin; SREBP2: sterol regulatory element binding protein-2, Pgp: P-glycoprotein; CRT: calreticulin; d: doxorubicin; DHA: docosahexaenoic acid; EPA: eicosapentaenoic acid. [file 1476-4598-12-137-S5.tiff]
